# Mesenchymal stromal cells mitigate liver damage after extended resection in the pig by modulating thrombospondin-1/TGF-β

**DOI:** 10.1038/s41536-021-00194-4

**Published:** 2021-12-03

**Authors:** Sandra Nickel, Sebastian Vlaic, Madlen Christ, Kristin Schubert, Reinhard Henschler, Franziska Tautenhahn, Caroline Burger, Hagen Kühne, Silvio Erler, Andreas Roth, Christiane Wild, Janine Brach, Seddik Hammad, Claudia Gittel, Manja Baunack, Undine Lange, Johannes Broschewitz, Peggy Stock, Isabella Metelmann, Michael Bartels, Uta-Carolin Pietsch, Sebastian Krämer, Uwe Eichfeld, Martin von Bergen, Steven Dooley, Hans-Michael Tautenhahn, Bruno Christ

**Affiliations:** 1 Department of Visceral, Transplant, Thoracic and Vascular Surgery, University of Leipzig Medical Center, Leipzig, Germany; 2 Division of General, Visceral and Vascular Surgery, University Hospital Jena, Jena, Germany; 3 Systems Biology and Bioinformatics, Leibniz Institute for Natural Product Research and Infection Biology – Hans Knöll Institute (HKI), Jena, Germany; 4 Lehrstuhl für Bioinformatik, Friedrich-Schiller-Universität Jena, Jena, Germany; 5 Department of Molecular Systems Biology, Helmholtz Centre for Environmental Research - UFZ, Leipzig, Germany; 6 Institute of Transfusion Medicine, University of Leipzig Medical Center, Leipzig, Germany; 7 Institute of Biology, Animal Ecology, Martin-Luther-University Halle-Wittenberg, Halle, Germany; 8 Department of Orthopedics, Trauma and Plastic Surgery, University of Leipzig Medical Center, Leipzig, Germany; 9 Department of Medicine II, Molecular Hepatology Section, Medical Faculty Mannheim, Heidelberg University, Mannheim, Germany; 10 Faculty of Veterinary Medicine, Department of Forensic and Toxicology, South Valley University, Qena, Egypt; 11 Large Animal Clinic for Surgery, University of Leipzig, Leipzig, Germany; 12 Department of Anaesthesiology and Intensive Care, University Hospital Leipzig, Leipzig, Germany; 13 Institute of Biochemistry, Leipzig University, Leipzig, Germany; 14 Research Programme “Else Kröner-Forschungskolleg AntiAge”, Jena University Hospital, 07747 Jena, Germany; 15 Present Address: Klinik für Allgemein-, Viszeral- und Thoraxchirurgie, HELIOS Park-Klinikum Leipzig, Leipzig, Germany

**Keywords:** Regenerative medicine, Mesenchymal stem cells

## Abstract

Post-surgery liver failure is a serious complication for patients after extended partial hepatectomies (ePHx). Previously, we demonstrated in the pig model that transplantation of mesenchymal stromal cells (MSC) improved circulatory maintenance and supported multi-organ functions after 70% liver resection. Mechanisms behind the beneficial MSC effects remained unknown. Here we performed 70% liver resection in pigs with and without MSC treatment, and animals were monitored for 24 h post surgery. Gene expression profiles were determined in the lung and liver. Bioinformatics analysis predicted organ-independent MSC targets, importantly a role for thrombospondin-1 linked to transforming growth factor-β (TGF-β) and downstream signaling towards providing epithelial plasticity and epithelial-mesenchymal transition (EMT). This prediction was supported histologically and mechanistically, the latter with primary hepatocyte cell cultures. MSC attenuated the surgery-induced increase of tissue damage, of thrombospondin-1 and TGF-β, as well as of epithelial plasticity in both the liver and lung. This suggests that MSC ameliorated surgery-induced hepatocellular stress and EMT, thus supporting epithelial integrity and facilitating regeneration. MSC-derived soluble factor(s) did not directly interfere with intracellular TGF-β signaling, but inhibited thrombospondin-1 secretion from thrombocytes and non-parenchymal liver cells, therewith obviously reducing the availability of active TGF-β.

## Introduction

The pleiotropic actions of mesenchymal stromal cells (MSC) rely on their differentiation, migratory, and secretory potential, enabling them to target sites of tissue injury, in order either to provide cellular and functional substitution or to support self-regeneration of the injured tissue^1,2^. In the liver, MSC attenuated fatty liver diseases^3,4^, acute liver failure after acetaminophen^5,6^, or d-galactosamine intoxication^7^, as well as liver fibrosis and cirrhosis^8^. MSC improved liver function after extended partial hepatectomies (ePHx) in rodent animal models by ameliorating damage and supporting regeneration of the residual liver^9,10^. This is clinically relevant, as partial liver resection is the only cure for patients with liver tumors. Extended resections of more than 70% of the liver mass are frequently performed to achieve tumor-free resection margins rendering the residual liver with a critical high regenerative and functional demand until restoration of organ volume and function^11,12^. In connection with pre-surgically existing liver diseases, prior chemotherapy, surgical trauma, inflammation, and the high regenerative requirements, ePHx may cause organ dysfunction and finally failure with fatal consequences for the patient. Although factors associated with the patient (e.g., physical state, age, and gender), liver-related co-morbidities, and the complexity of surgery represent risk factors, no clear definition of post-hepatectomy liver failure (PHLF) is available based on standard clinical criteria^13^. Most commonly, liver surgeons apply the “50–50” rule, defined as prothrombin time <50% and serum bilirubin >50 µmol/L, as an early predictor of PHLF^14^, a largely empirical approach. As a consequence of liver failure, two major problems arise: first, encephalopathy and multi-organ dysfunction partly due to reduced detoxification by the liver concomitant with the accumulation of (neuro)toxic compounds and, second, the reduction of blood coagulation due to the decrease of the hepatic synthesis of plasma proteins involved in the coagulation cascade. Thus, factors involved in the regulation of hemostasis play a role in the outcome of post-hepatectomy survival, which is substantiated by the suggestion to include the prothrombin time in addition to the 50–50 criteria into a LASSO model to predict PHLF^15^. Similarly, besides factors as mentioned above, the platelet count has been included in a model to predict post‐surgery mortality in patients suffering from liver cirrhosis^16^, suggesting that thrombocytes (THCs) may critically be involved in post-surgery liver function. Indeed, platelet-derived factors were identified as attenuators of liver regeneration after partial hepatectomy comprising high post-operative thrombospondin-1 (THBS1) as a negative predictor of surgical outcome and impairment of liver regeneration^17^. Mechanistically, THBS1 was suggested to activate transforming growth factor-β (TGF-β)^18,19^, a prominent inhibitor of hepatocyte proliferation and mediator of parenchymal plasticity and epithelial-mesenchymal transition (EMT)^20,21^.

The rodent liver is different from humans and it remains open whether MSC support after ePHx may be clinically relevant. Moreover, mechanisms of MSC actions in the context of liver resection are largely unknown. In the pig model, with liver anatomy and physiology comparable to humans, we have shown that MSC treatment supported circulatory maintenance by preventing kidney injury after 70% liver resections, thus indicating improvement of multiple organ damage associated with liver surgery^22^. In the present study, in order to identify mechanisms involved, we anticipated that MSC prevented multiple organ dysfunction by similar or same mechanisms in different organs and further assumed that analysis of different organs would unravel such potentially shared mechanisms. We determined transcriptomic profiles in the liver and lung without and with MSC treatment. Joint bioinformatics analysis predicted that MSC may modulate the THBS1/TGF-β signaling axis.

## Results

### MSC improve liver function after extended resection

**Fig. 1:**
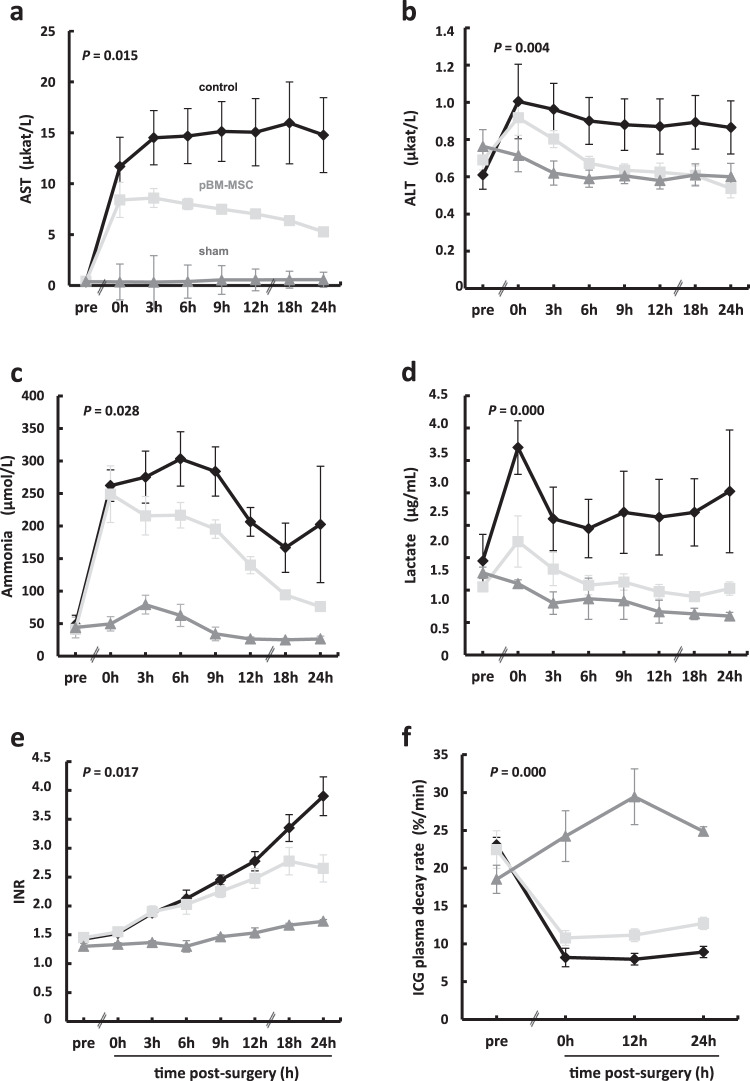
Time course of serum changes in liver function parameters during 24 h after extended liver resection. **a** Aspartate aminotransferase (AST), **b** alanine aminotransferase (ALT), **c** ammonia, **d** lactate, **e** international normalized ratio (INR), and **f** indocyanine green (ICG) clearance rate in control (ePHx only) and MSC-treated (ePHx and pBM-MSC) animals. Values shown are means ± SD from three animals in the sham and from four animals each in the control and the pBM-MSC treatment group. Statistically significant differences between control and MSC-treated animals, according to the Bonferroni post hoc test following general linear model (GLM), are defined at the *P*-level as indicated. For the GLM analysis, STATISTICA 8.0 (StatSoft, Tulsa, OK, USA) was used.

Besides the surgical trauma, ePHx causes functional impairment as demonstrated in control animals (ePHx only) by changes of hepatic parameters, including increases in serum aspartate aminotransferase (AST), alanine aminotransferase (ALT), ammonia, lactate, or international normalized ratio (INR). All these parameters are improved by the intravenous (*V. jugularis interna*) transfusion of porcine MSC from bone marrow (pBM-MSC). Also, the indocyanine green (ICG) clearance rate was higher in MSC-treated animals as compared to controls without MSC treatment, indicating a general functional improvement of the liver by the MSC (Fig. 1a–f).

### Analysis of the combined MSC treatment-associated regulatory module

Besides impairment of liver function, ePHx causes damage of the kidneys^22^, suggesting surgery-induced multi-organ dysfunction. As both liver and kidney functions were improved by systemic treatment with pBM-MSC, cells may act on different organs via common mechanisms. Therefore, we analyzed global gene expression changes in the liver, where we already verified organ damage, and in the lung, where we anticipated, but not yet verified organ damage, with and without pBM-MSC treatment, using bioinformatics tools (cf. “Methods”, for gene expression analyses and identification of regulatory modules).

**Fig. 2:**
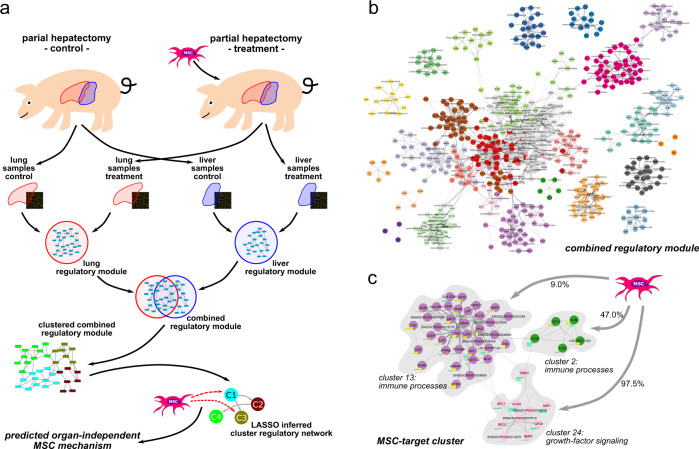
Schematic workflow for the prediction of organ-independent molecular MSC targets. **a** Gene expression was measured in lung and liver samples after partial hepatectomy of control and MSC-treated pigs. ModuleDiscoverer was used to identify organ-specific regulatory modules of the response to MSC treatment. Topological clustering of the combined regulatory module was performed to identify clusters of proteins with similar biological functions. Based on the average cluster expression profiles for lung and liver samples, and MSC treatment as additional perturbation, we computed a cluster regulatory network. The predicted relations between MSC and the clusters resemble potential mechanisms of MSC action. **b** Combined regulatory module composed of the union of the lung-specific and the liver-specific regulatory modules. For reading of individual protein members in the 29 clusters (please cf. Supplementary Data 1). **c** The STRING-network outlining all proteins that belong to clusters predicted as targeted by MSC. Cluster membership of proteins is color-coded. For each protein, log2-FC for the liver (left) and lung (right) of the associated gene is shown as chart next to the protein. Up-/downregulation is colored yellow/turquoise, respectively.

By the gene array measurements, we first identified the differentially expressed genes (DEGs) in control and treatment samples from the liver and lung individually. Based on the two sets of DEGs and the organism-specific high-confidence protein–protein interaction network (PPIN) provided by STRING, we then applied the ModuleDiscoverer^23^ algorithm to identify organ-specific regulatory modules representative for the molecular characterization of the response to MSC treatment after ePHx. By unification of these two regulatory modules, a combined regulatory module was created containing regulatory module complementing information about molecular pathways and biological processes from both organ-specific modules as well as the organ-independent proteins affected (overlapping nodes). Based on topological clustering, we then identified clusters of proteins involved in similar biological functions and pathways. Finally, we used a LASSO-based network inference approach to identify a cluster regulatory network (CRN), including MSC treatment as additional perturbation. From the predicted edges between MSC treatment and protein clusters, we extracted hypotheses about the molecular mechanism of the observed beneficial MSC effects (Fig. 2a).

The final MSC treatment-associated regulatory module was created by unification of the two organ-specific regulatory modules (cf. “Methods”, Identification of organ-specific regulatory modules using ModuleDiscoverer). The unified module contains 640 proteins connected by 4318 relations. Forty-one proteins are shared by liver- and lung-specific regulatory modules. Topological clustering based on the general topological overlap measure^24^ reveals 29 protein clusters (Fig. 2b), which are associated with different biological processes according to a GeneOntology enrichment analysis (Supplementary Data 1, SubModule Enrichment Analysis). For each of these 29 protein clusters, we calculated a representative expression value based on the median of the expression values of all proteins within the respective cluster. A heatmap of the scaled expression values for each cluster shows that the samples cluster mostly by treatment, not by organ (Supplementary Fig. 1).

### Inference and analysis of a CRN

Protein clusters specifically associated with MSC treatment were identified by inferring a CRN (cf. “Methods,” LASSO inferred cluster regulatory network). We found that the treatment variable (MSC stimulus) was connected to cluster 24 in 97.5%, cluster 2 in 47.0%, and cluster 13 in 9.0% of all runs (Fig. 2c). These clusters are significantly enriched (Supplementary Data 1, Submodule Enrichment Analysis) with targets associated to fibroblastic growth factor receptor signaling, focal adhesion, and extracellular matrix-receptor interaction in cluster 24, the innate immune response, detection of external biotic stimuli in cluster 2, as well as antigen processing and presentation of peptide or polysaccharide antigens via major histocompatibility complex class II and cell activation in cluster 13. This is shown in detail as a STRING-based network in Fig. 2c. The top central proteins connecting the different clusters, i.e., those with the highest betweenness centrality measure, were THBS1, the thrombospondin receptor CD36, glypican-1, lymphocyte cytosolic protein-2, and the FYN proto-oncogene, Src family tyrosine kinase (FYN; ENSSSCP00000004769). Notably, THBS1, CD36, and FYN are all involved in regulation of tissue remodeling during growth and differentiation and are present in the two organ-specific regulatory modules. Because of its exposed position, THBS1 was assigned a major target of MSC treatment after ePHx. We extracted from these data the hypothesis that downregulation of THBS1 by MSC might improve organ function.

### pBM-MSC attenuate resection-induced increases in plasma and liver THBS1 and TGF-β

**Fig. 3:**
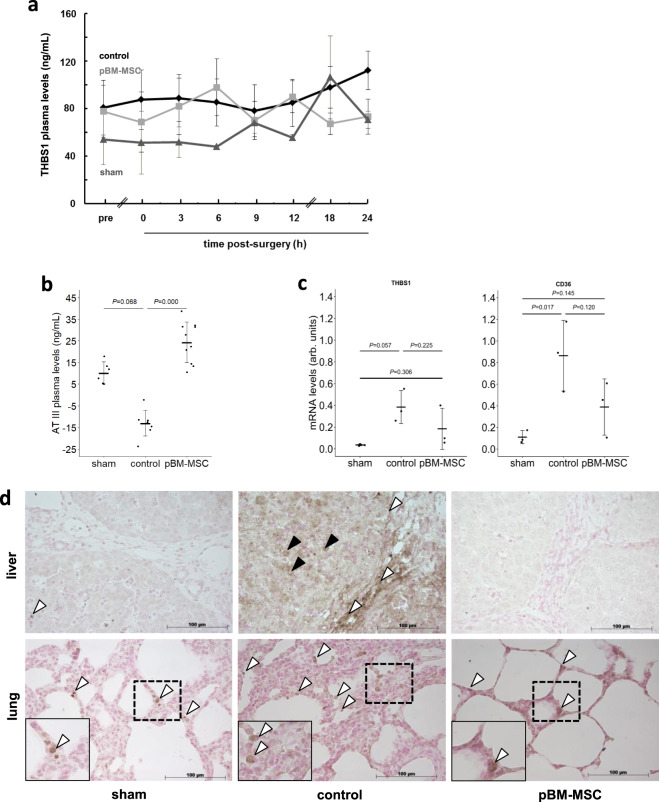
Plasma levels and expression of THBS1 after ePHx with and without MSC treatment. Plasma samples from five animals in each of the control and MSC-treated group, and three in the sham group were collected at the indicated time points, and **a** THBS1 and **b** Antithrombin III levels determined by ELISA (means ± SD). The Kruskal–Wallis test was performed (*P* *=* 0.0001) and significant differences between the groups were evaluated by post hoc Bonferroni test. **c** THBS1 and CD36 mRNA levels were determined in liver samples of three animals in each group at 24 h after hepatectomy. PCR products were quantified using PPIA (Peptidylprolyl Isomerase A) for normalization. Values are given as means ± SD (*n* = 3; statistics: one-way ANOVA) as calculated from the representative gels shown in Supplementary Fig. 2. **d** Immunohistochemical detection of THBS1 (arrowheads) in the liver (upper images) and lung (lower images) of sham (left), control (middle), and MSC-treated (right) pigs at 24 h after liver surgery. Images show ×20 original magnifications of representative organ tissue slices from three pigs per group. In the liver, staining is located in the portal tracts lining blood vessels and bile ducts (white arrowheads) and in the parenchyma (black arrowheads). In the lung, THBS1 is expressed in cells lining the alveolae (white arrowheads; the insets show higher magnifications (computed) of the dashed areas).

In controls, THBS1 plasma levels increase during the 24 h post surgery. In animals treated with pBM-MSC, THBS1 followed a likewise oscillatory time course and reaches levels equal to sham-treated animals after 24 h. Levels are 1.5-fold higher in control as compared to MSC-treated animals (Fig. 3a). Twenty-four hours post surgery, antithrombin III (AT III), an inhibitor of thrombin-activated THBS1 release from platelets^25^, is decreased in the plasma of control animals with ePHx, as compared to pBM-MSC-treated pigs, which display levels in the range of sham-treated animals (Fig. 3b). As compared to livers of sham-treated animals, mRNAs of THBS1 and its receptor CD36 are elevated after ePHx by about 15- and 10-fold, respectively. These levels are reduced by 50% upon pBM-MSC treatment, which is similar to those from sham-treated animals (Fig. 3c and cf. Supplementary Fig. 2). The elevation of THBS1 in liver after ePHx is confirmed at the protein level immunohistochemically, comparing controls with sham-treated animals. THBS1 is found in non-parenchymal areas of the portal tracts and in parenchyma. pBM-MSC treatment decreases THBS1 to nearly undetectable levels (Fig. 3d, upper images). We found similar results in lung tissue (Fig. 3d, lower images).

**Fig. 4:**
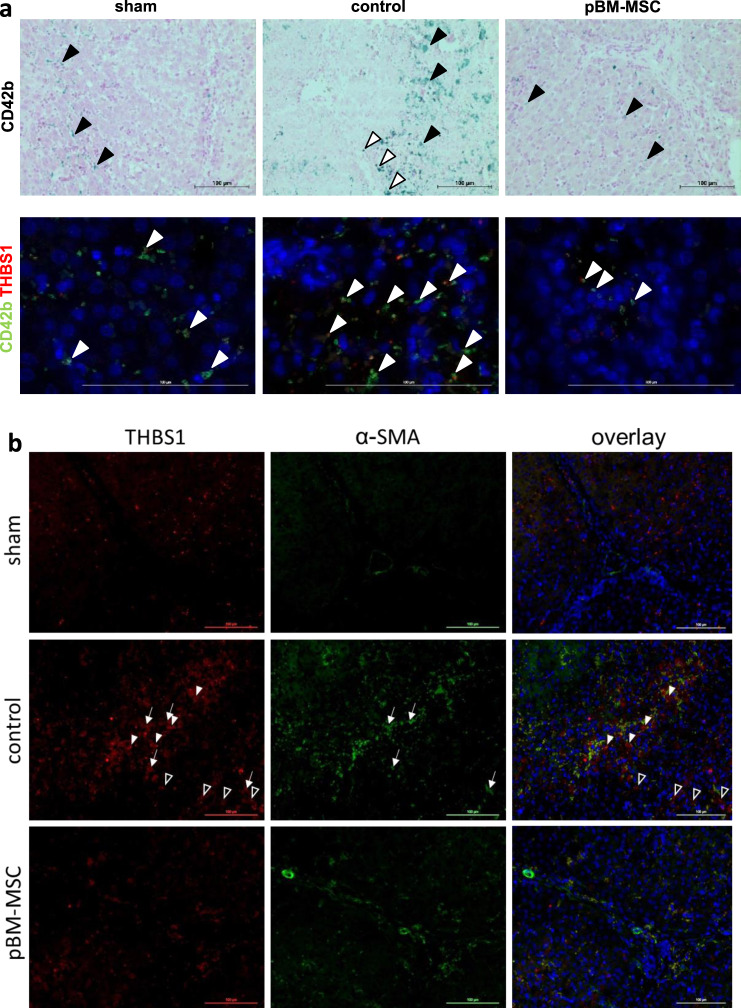
MSC treatment attenuates the hepatic increase in platelets and activated HSC after ePHx. **a** Detection of thrombocytes (CD42b, upper panels) and colocalization of thrombocytes with THBS1 (green and red fluorescence, resp., lower panels) in livers of sham, control, and MSC-treated pigs. In the upper panels, CD42b is marked in the parenchyma (black arrowheads) and occasionally lining the septae (white arrowheads). In the lower panels, CD42b mainly colocalizes with THBS1 in the parenchyma (white arrowheads). Original magnification: ×20 (upper) and ×63 (lower). Scale bar, 100 µm. **b** Immunofluorescent detection of THBS1 and α-SMA (as a marker of activated HSC) in livers of sham, control, and MSC-treated pigs; THBS1 (left panels, red), α-SMA (middle panels, green), and overlay with DAPI co-stains (right panels). Tissue samples were taken at 24 h after ePHx. THBS1 is marked in the septae (white arrowheads) and in the parenchyma (black arrowheads). Partial colocalization of THBS1 and α-SMA is observed both in septae and parenchyma (white arrows). Images are representative for each three slices out of the organs from three different animals per group. Original magnification, ×20. Scale bar, 100 µm.

To delineate, whether THCs, a major source of THBS1^26^, are enriched in the liver after ePHx, we used CD42b for visualization by immunohistochemistry. Extended liver resection markedly increases THCs in the liver localizing mainly in the parenchyma without preferential periportal or pericentral enrichment. Occasional staining is also visible along the septae. This increase is attenuated by pBM-MSC treatment down to levels comparable with sham-treated animals (Fig. 4a). The co-stain of CD42b with thrombospondin, the latter of which localizes in the septae and the parenchyma (cf. Fig. 3d), is mainly restricted to the parenchyma designating THCs a major source of parenchymal, but not septal THBS1 (Fig. 4a and Supplementary Fig. 3). Detection of THCs using the CD42b antibody in the kidney also shows marked increase after ePHx as compared with sham-treated animals. Again, this increase is abrogated by treatment with pBM-MSC (Supplementary Fig. 4). Staining in controls demarks glomerular and tubular localization of THCs. Yet, only parts of the tubular epithelia are stained, consistent with our previous finding that kidney damage after ePHx mainly affected proximal tubulus epithelia^22^.

To demonstrate whether activated hepatic stellate cells (HSCs), as shown in mice before by another group^19^, were a source of THBS1, tissue sections were co-stained for THBS1 and α-smooth muscle actin (α-SMA). Although in control animals both THBS1 and α-SMA are increased, treatment with pBM-MSC attenuates this increase down to the levels in livers of sham-treated animals. Partial THBS1 and α-SMA colocalization in septae and parenchyma indicates that activated HSC and cells residing in the connective tissue of the septae are likely another prominent source of THBS1 different from THCs (Fig. 4b). THBS1 does not colocalize with CD31 on endothelial cells indicating that these are likely not involved (Supplementary Fig. 5).

These experimental findings corroborate the predictions from the bioinformatics analysis, i.e., MSC treatment attenuates surgery-induced THBS1 in the liver and lungs.

**Fig. 5:**
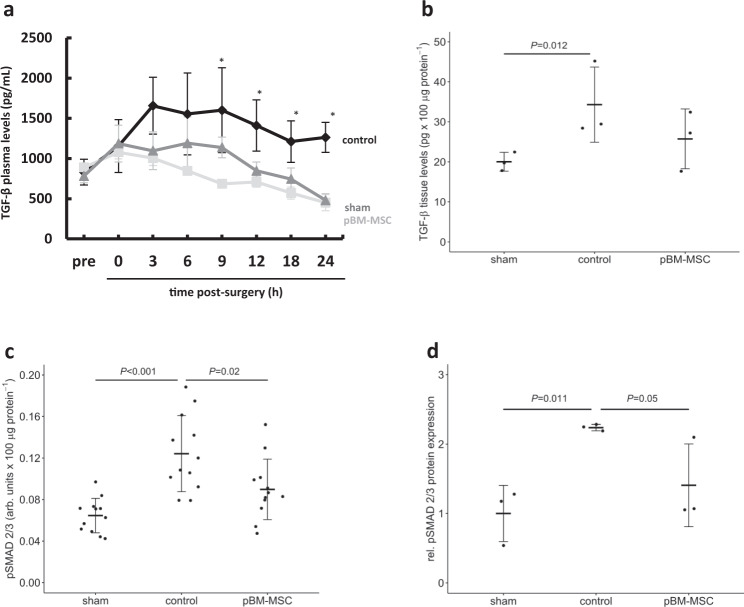
Inhibition of the ePHx-induced increase of TGF-β and TGF-β signaling in the liver by pBM-MSC. **a** TGF-β plasma levels were determined by ELISA in control, sham-, and MSC-treated animals. Values shown are means ± SD from three animals in each group. The Kruskal–Wallis test was performed and significant differences between the groups were evaluated by post hoc Bonferroni test (*P ≤ 0.05). **b** TGF-β was determined in liver tissue samples at 24 h after ePHx by ELISA in sham, control, and MSC-treated animals as indicated. Values are means ± SD from two different tissue samples out of three animals in each group. The Kruskal–Wallis test was performed and significant differences between the groups were evaluated by post hoc Bonferroni test. **c** The phosphorylation of Smad 2/3, indicating active TGF-β signaling, was determined in liver tissue samples at 24 h after ePHx by ELISA in sham-, control-, and MSC-treated animals. Values are means ± SD from two different tissue samples out of three animals in each group. **d** Results from **c** are confirmed by western blot analysis of pSmad 2/3 protein levels. Protein expression was compared to total Smad 2/3 levels and normalized to reference protein expression (GAPDH); values shown are relative to the expression in sham-treated animals, which was set arbitrarily to 1. Values are given as means ± SEM (*n* = 3; statistics: one-way ANOVA) as calculated from the representative immunoblots shown in Supplementary Fig. 6.

TGF-β can become activated by release from its extracellular matrix-deposited latent form (LTGF-β) upon interaction of THBS1 with the latency-associated peptide of the LTGF-β^27^. Consistently, ePHx increases TGF-β plasma levels in control animals by more than twofold over pre-surgery levels and levels remain elevated for the following 24 h. In pBM-MSC-treated animals, TGF-β levels are also slightly increased over pre-surgery levels, but in the same range as those from sham-operated animals, and these levels decreased continuously during the 24 h observation time post surgery (Fig. 5a). In addition, high TGF-β plasma levels in untreated ePHx control animals correspond to significantly higher TGF-β tissue levels (Fig. 5b) and signaling pathway activation in the liver, as shown by phosphorylation of Smad 2/3 with an enzyme-linked immunosorbent assay (ELISA). pSmad 2/3 is increased about twofold (Fig. 5d) and decreased at 24 h post-ePHx in the pBM-MSC treatment group as confirmed by western blotting using an anti-phospho-Smad 2/3 antibody (Fig. 5d and cf. Supplementary Fig. 6).

### pBM-MSC treatment attenuates ePHx-induced tissue plasticity and EMT in the liver

**Fig. 6:**
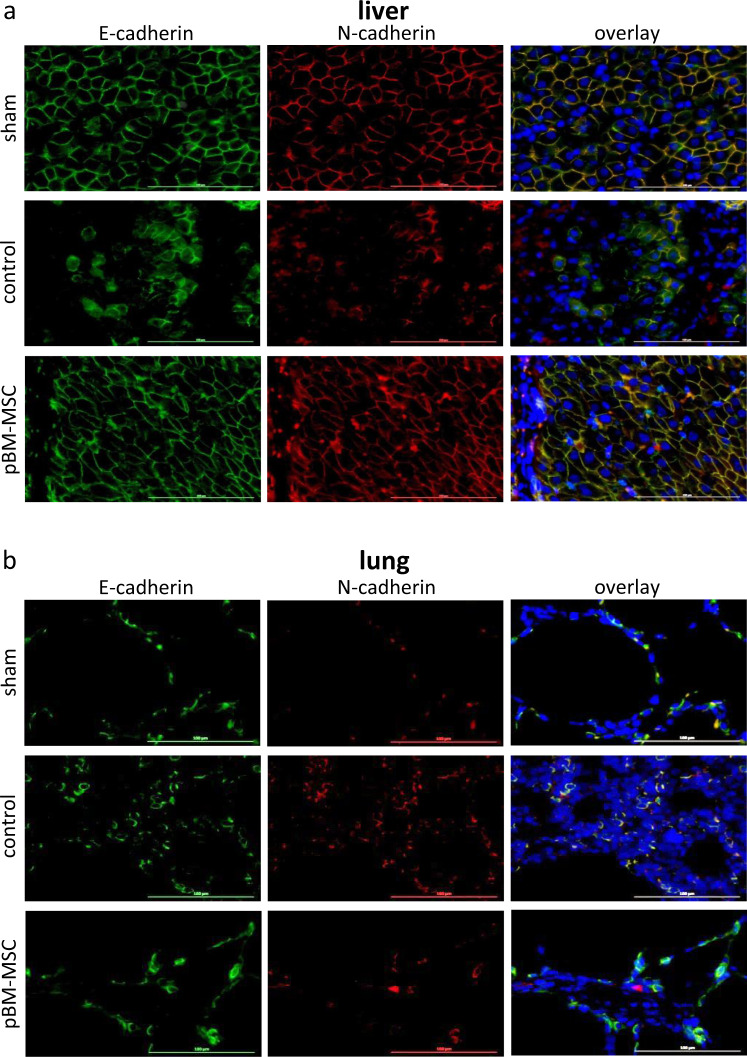
Protection of adherens junctions from damage after ePHx by MSC treatment. **a** Immunofluorescent detection of E-cadherin and N-cadherin in the liver and **b** lung of sham-, control-, and MSC-treated pigs; E-cadherin (left panels, green), N-cadherin (middle panels, red), and overlay with DAPI (blue) co-stains (right panels). Tissue samples were taken at 24 h after ePHx. As compared to sham-treated animals, E- and N-cadherin fade away after resection (upper vs. middle panels) in the livers, whereas in the lung, N-cadherin emerges. Both signs of tissue damage are attenuated by the treatment with pBM-MSC (middle vs. bottom panels). Images are representative for three slices out of the organs of three animals per group. Original magnification, ×20.

THBS1 might promote epithelial cell plasticity required for tissue remodeling after injury, either by signaling via its receptors CD36 and/or CD47, or via activation of TGF-β^28^. In livers of sham-treated animals, the adherens junction proteins E- and N-cadherin continuously colocalize in periportal hepatocytes. After ePHx, this pattern changes and expression is confined to small patches or strings of hepatocytes adjacent to large regions without expression. pBM-MSC treatment preserves the continuous pattern as observed in the sham-operated animals (Fig. 6a). In the lung, N-cadherin is hardly detectable in sham-treated animals, but is co-expressed with E-cadherin after ePHx. This is consistent with previous findings that N-cadherin is only expressed in the setting of TGF-β-mediated EMT that is occurring upon lung injury^29^. We conclude that expression of N-cadherin in controls indicates epithelial damage in the lung after ePHx, which is attenuated by the pBM-MSC treatment (Fig. 6b).

ZO-1 is expressed ubiquitously in the livers of sham-treated animals at the basolateral membranes of hepatocytes labeling the bile canaliculi. In controls with ePHx only, ZO-1 expression is largely restricted to small areas in the parenchyma. Except for expression in these singular patches, ZO-1 is barely detectable. In livers of pBM-MSC-treated animals, ZO-1 and E-cadherin expression are preserved ubiquitously in the parenchyma similarly to expression in sham-treated animals. In the lungs of sham-treated animals, ZO-1 is expressed continuously along the alveolar epithelia. Extended PHx causes distortion of this pattern revealing interruption of continuous expression by gaps void of ZO-1 expression. Treatment with pBM-MSC preserves continuity (Supplementary Fig. 7).

**Fig. 7:**
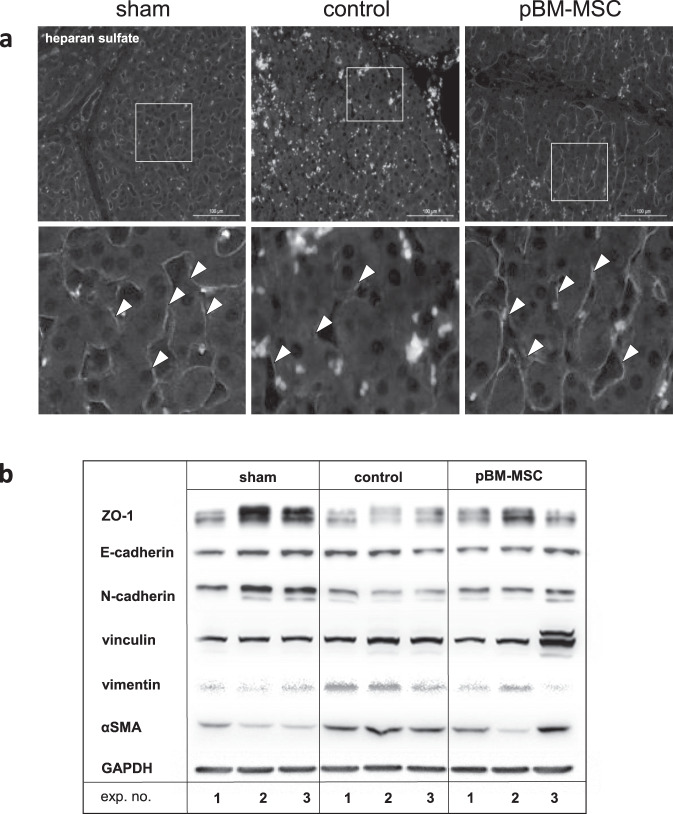
MSC treatment protects the liver from EMT after ePHx. **a** Immunofluorescent detection of heparan sulfate (arrowheads) in the liver of sham, control, and MSC-treated pigs. Tissue samples were taken at 24 h after ePHx. Heparan sulfate is hardly detectable after resection (middle panels) in comparison with sham-treated animals (left panels). Treatment with pBM-MSC preserves expression of heparan sulfate (right panels). Lower panels show computed image magnifications of the boxes drawn in the original pictures (upper panels). Images are representative for three slices out of the organs of three animals per group. Original magnification, ×20. **b** The surgery-induced decrease/increase of expression of epithelial (ZO-1, E-cadherin, N-cadherin) and mesenchymal (vinculin, vimentin, α-SMA) markers is confirmed by western blotting. Original blots of three animals in each group are shown instead of quantitative analysis because of the high inter-individual variability.

Dynamics and plasticity of cell adhesions are based, besides others, on the crosstalk between growth factor signaling and the extracellular matrix^30^. Here we investigated heparan sulfate (HS) as a surrogate for the response of the extracellular matrix to ePHx. Expression in the liver decreases after surgery, which is indicative for functional perturbations. This can be reduced by MSC treatment, maintaining HS expression at similar levels present in sham-treated animals (Fig. 7a). Further, the decrease of HS expression is paralleled by decreased epithelial (E-, N-cadherin, ZO-1) and increased mesenchymal (vinculin, vimentin, and αSMA) markers, which are altogether reversed in part at least by treatment with pBM-MSC (Fig. 7b).

### pBM-MSC inhibit THBS1 secretion and indirectly TGF-β signaling

**Fig. 8:**
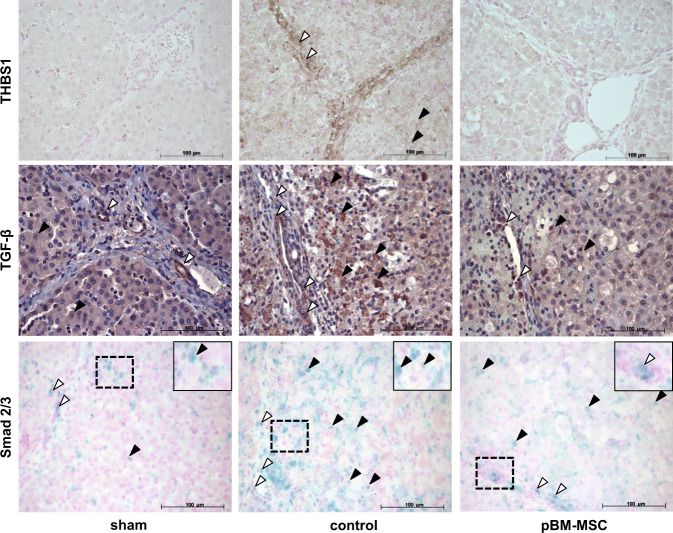
MSC treatment attenuates the ePHx-dependent increase in THBS1, TGF-β, and activated Smad 2/3 staining. Immunohistochemical detection of THBS1 (brown, upper panels), TGF-β (brown, middle panels), and Smad 2/3 (blue, bottom panels) in livers of sham- (left column), control- (middle column), and MSC-treated (right column) pigs. Tissue samples were taken at 24 h after resection. Images are representative for three slices out of the organs of three animals per group. White and black arrowheads indicate portal tract and parenchymal localization, respectively. Original magnification, ×20. The insets show higher magnifications (computed) of the dashed areas.

THBS1 might induce injury-mediated cell plasticity/EMT by activation of TGF-β. Such a causal relationship should be evident, among others, by colocalization of THBS1, TGF-β, and Smad activation in the liver. In sham-treated animals, THBS1 is hardly visible. TGF-β stain is found in cells of the portal tracts and distributed homogeneously in the parenchyma. Activation of TGF-β signaling is rarely detectable in the parenchyma of livers from sham-operated animals, as evident by the lack of nuclear staining upon anti-Smad 2/3 antibody usage (Fig. 8, left panels). Liver resection causes a marked increase of THBS1, TGF-β, and nuclear phospho-Smad 2/3, both in portal tracts and in the parenchyma (Fig. 8, middle panels). In addition, the surgery-induced increase in THBS1 colocalizes with the activation of TGF-β signaling, which is clearly mitigated in samples from animals that underwent pBM-MSC treatment (Fig. 8, right panels).

To study if pBM-MSC effects on TGF-β signaling in hepatocytes are direct or indirect, we performed in vitro experiments using co-cultures of pBM-MSC and primary porcine hepatocytes (pHCs). After stimulation with TGF-β, pSmad 2/3 increases both in pBM-MSC and hepatocytes when cultured alone each at a cell density of 200,000 cells/dish (one well in a 6-well plate). When cultured together at a density of 100,000 of each cell type per dish, the increase is half of the additive effect of the cells when cultured separately indicating that pBM-MSC exhibited no inhibitory action. Similarly, treatment of pHCs with conditioned medium (CM) derived from pBM-MSC does not attenuate TGF-β-induced pSMAD 2/3 in the hepatocytes. Thus, it may be assumed that neither MSC nor MSC-derived factors directly inhibit TGF-β signaling in pig hepatocytes. To substantiate this conclusion, the nuclear translocation of Smad 2/3 was monitored by immunocytochemistry in co-cultures of MSC with primary pHCs using an antibody detecting both the native and the phosphorylated Smad 2/3. When cultured separately, both pBM-MSC and pHC display marginal nuclear localization of Smad 2/3, which is significantly elevated after treatment with 1 ng/mL TGF-β. When co-cultured, TGF-β treatment also increases nuclear localization of Smad 2/3 in both pHC and pBM-MSC (Supplementary Fig. 8), substantiating that pBM-MSC do not directly affect TGF-β signaling in pHCs. Similarly, human bone marrow-derived MSC (hBM-MSC) do not ameliorate the TGF-β-induced perturbation of cell–cell contacts in co-cultures with the epithelial cell line MDCK II as evidenced by the decrease in ZO-1 expression (Supplementary Fig. 8).

**Fig. 9:**
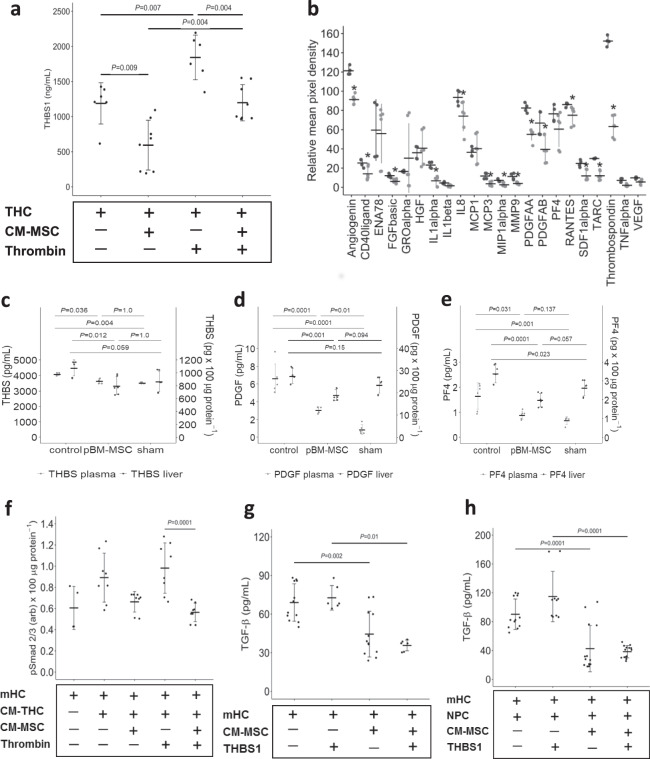
Factors derived from pBM-MSC inhibit THBS1 secretion from thrombocytes. **a** THBS1 secretion from thrombocytes (THC) into the supernatant medium was determined by ELISA after 10 min of culture either without or in the presence of conditioned medium derived from MSC (THC + CM-MSC). Where indicated, cultures were treated with thrombin (10 U/mL) to stimulate THBS1 secretion. Values are means ± SEM (*n* = 6 for THC, *n* = 8 for THC + CM-MSC; statistics: one-way ANOVA; post hoc Bonferroni test) and are significantly different at the *P*-levels as indicated. **b** Secretion of proteins from THC into the supernatant medium was determined by cytokine array analysis after 10 min of culture either without (dark gray; two different cultures analyzed in duplicate) or in the presence (light gray; three different cultures analyzed in duplicate) of conditioned medium derived from MSC. *Values are significantly different at the *P* ≤ 0.05 level (Student’s *t*-test for pairwise comparison of each single cytokine w vs. w/o conditioned medium). **c** THBS1, **d** PDGF-AB, **e** PF4 levels in porcine plasma (light gray dots) and liver tissue (dark gray dots) were determined in duplicate by ELISA at 24 h post surgery in control animals, in animals treated with pBM-MSC, and in sham-treated animals. Values shown in (**c**–**e**) are means ± SEM from three different animals in each group and are significantly different at the *P*-level ≤ 0.05 as indicated (statistics in addition to Johnson transformation: one-way ANOVA, post hoc Bonferroni test). **f** Isolated mouse hepatocytes (mHCs) were treated with supernatants from THC (CM-THC) as described in (**a**) for 1 h and pSmad 2/3 quantified in the hepatocytes by ELISA. Where indicated, THC were pre-treated with thrombin (10 U/mL) to stimulate THBS1 secretion. Values represent means ± SD from three or more independent cell cultures, each analyzed in duplicate. Statistics in addition to Johnson transformation: one-way ANOVA, post hoc Bonferroni test. **g** Isolated mouse hepatocytes (mHCs) were treated with THBS1 (1 µg/mL) as indicated and incubated for 1 h with or without conditioned medium derived from MSC. TGF-β was determined by ELISA as described in the “Methods” section. **h** Experiments were performed as described under (**g**), except that non-parenchymal liver cells (NPCs) were included where indicated. Values in (**g**) and (**h**) represent means ± SD from three or more independent cell cultures, each analyzed in duplicate. Statistics: one-way ANOVA, post hoc Bonferroni test.

Thus, MSC are not directly interfering with TGF-β signaling or action. The remaining option is inhibiting the increase of the available amount of active TGF-β from the extracellular matrix-deposited latent form of the cytokine, where the identified upregulated THBS1 level is a promising candidate. We have shown that THCs are a major source of upregulated THBS1 in the liver after ePHx (cf. Fig. 4). We therefore investigated, whether hBM-MSC can attenuate THBS1 secretion from THC and incubated human THCs with or without CM from hBM-MSC (CM-MSC). Indeed, CM-MSC significantly decrease THBS1 levels to about one half. This is as well the case, when we stimulated platelets with 10 U/mL thrombin; here, THBS1 secretion from thrombin-stimulated THC is significantly lowered by 35% in the presence of CM-MSC (Fig. 9a). Interestingly, platelet secretion inhibition does not only affect THBS1, but also other secretory proteins known to be contained in THC α-granules^31^ comprising, besides others, angiogenin, FGFbasic, IL1α, IL8, MCP3, PDGF, and SDF1α, as determined by cytokine array analysis (Fig. 9b). We assume from these data that MSC-derived factors attenuate secretion from THC α-granules. To substantiate this assumption in our porcine model, we determined plasma levels of THBS1, PDGF, and PF4, prominent factors secreted by activated THCs. The surgical procedure increases levels of the three proteins significantly over values in sham-operated animals, which is attenuated by treatment with pBM-MSC. Inhibition of the surgery-induced increase of THBS1, PDGF, and PF4 is also observed in liver tissue (Fig. 9c–e). Thus, MSC treatment inhibits secretion from platelets in vitro and very likely also in vivo.

Further, attenuation of secretion may be not targeted towards platelets only, but rather represents a general mechanism of MSC action. To test this assumption, we chose human umbilical vein endothelial cells (HUVECs) known to secrete THBS1 autonomously^32^. THBS1 increases over time by about fivefold, which is reduced to about one half in co-cultures with hBM-MSC, or by MSC-derived CM, indicating the involvement of as-yet unknown soluble factor(s). Immunocytochemical detection of THBS1 in the HUVEC reveals that culture in MSC-CM blunted the synthesis of THBS1 both on RNA and protein levels as verified by reverse-transcription PCR (RT-PCR) and western blotting (Supplementary Fig. 9). These data demonstrate that MSC impair secretion of THBS1 also as consequence of expression inhibition.

To demonstrate a causal relationship between the levels of THBS1 secretion by THC and the strength of TGF-β signaling in hepatocytes, we treated mouse hepatocyte (mHC) cultures with CM from human THC (CM-THC), pre-treated or not with CM-MSC. Hepatocytic pSmad is augmented by CM-THC. This effect is slightly but not significantly attenuated by pre-treatment of THC with CM-MSC. When CM-THC is used from 10 U/mL thrombin pre-stimulated THC, the pSmad inhibiting effect of CM-MSC pre-treatment becomes significant (Fig. 9f). Thus, CM-MSC-mediated inhibition of TGF-β signaling in hepatocytes is mirrored by inhibition of THBS1 secretion from THC (cf. Fig. 9a), which is consistent with an interference with the THBS1/TGF-β axis by MSC treatment.

To directly prove whether CM-MSC inhibited the activation of TGF-β in hepatocytes, we incubated mHCs with CM-MSC with or without THBS1. Stimulation of hepatocytes alone with THBS1 does not increase TGF-β levels, indicating that hepatocytes are not a source for THBS1-mediated TGF-β activation. However, treatment with CM-MSC significantly decreases TGF-β levels in the medium consistent with the inhibition of hepatocytic secretion as seen likewise also with HUVEC and THC (Fig. 9g). Next, we included non-parenchymal cells (NPCs), known as the major source of TGF-β in the liver, as cellular root for TGF-β after treatment with THBS1. We co-cultured mHCs with NPC from the same livers, which increases TGF-β as compared with mHCs alone (Fig. 9h vs. 9g). This increase is slightly, yet not significantly, augmented by treatment with THBS1, in line with the THBS1-mediated provision of TGF-β by the NPC. Both the THBS1-stimulated and the -unstimulated TGF-β levels were significantly decreased by the treatment with CM-MSC (Fig. 9h). As ePHx increases THBS1 and TGF-β in the septae and in the hepatic parenchyma, which harbor a major fraction of NPC (cf. Fig. 8), these findings are consistent with the assumption that MSC may attenuate the secretion and/or activation of TGF-β in the liver indirectly by the inhibition of THBS1, thus reducing the availability of active TGF-β.

To substantiate these findings, we went back to the transcriptomic data gained from liver and lung, and analyzed combined upstream regulators affected by MSC treatment in both the lung and liver using Ingenuity Pathway Analysis (IPA). The analysis confirmed that in both organs TGF-β, but not THBS1 is attenuated by MSC treatment. Similarly, we observe downregulation of YAP1 in both organs, a regulator of size control and EMT after partial liver resection^33,34^. TGF-β target genes were visualized and reflect regulation in different subcellular compartments with different targets in both organs (Supplementary Fig. 10). Thus, the IPA corroborates in silico predictions from the network analysis and verifications in vivo that MSC attenuate EMT after ePHx likely by interference with THBS1-mediated availability of TGF-β.

## Discussion

Our experiments verified the systems biology-based prediction that treatment with pBM-MSC will ameliorate the increase of plasma and tissue THBS1 and TGF-β provoked by ePHx. As the stem cells were applied systemically, it is likely that the effect was provided by soluble factor(s), which we confirmed in vitro with isolated hepatocytes combined with NPC and THC. THBS1 and TGF-β are known mediators of hepatocyte plasticity and EMT in cells under stress and in advance of hepatocarcinogenesis^21,35^, which was confirmed here in the liver and lung after ePHx, and which was ameliorated by MSC treatment. This corroborated previous data showing that ePHx affects kidney epithelial integrity, which was also mitigated by pBM-MSC treatment^22^. It may hence be concluded that pBM-MSC protect epithelia from damage in multiple organs, thus generally supporting functional maintenance post surgery. As the beneficial effect of MSC are observed in the 24 h time frame after ePHx, which is likely too short for repair of surgery-induced tissue damage, we speculate that MSC rather prevent than repair damage. Mechanistically, this is in line in our approach with attenuating THBS1 secretion and downstream activation of latent TGF-β^36^, as well as TGF-β signaling in the liver and other organs. The mechanism of secretion inhibition by MSC may only be speculated upon facing the magnitude of pleiotropic actions of MSC, including secretion of soluble factors, exosomes, mitochondrial transfer, and cell–cell communication^37^. Yet, PGE_2_, a well-known MSC-derived factor featuring immunomodulatory functions, may be assumed to inhibit platelet secretion by the EP receptor-stimulated rise in intracellular cAMP, an antagonist of Ca^2+^-mediated secretion from platelets^38^. This hypothesis, however, needs experimental confirmation.

In THBS1-knockout mice, liver regeneration after PHx improves by the enhancement of proliferation and suppression of apoptosis, indicating a suppressor role of THBS1. This is likely due to activation of NPC- and THC-derived TGF-β, presumably an inhibitor of liver regeneration after partial hepatectomy^19^, which substantiates our data presented here. Also in patients, high THBS1 and TGF-β plasma levels correlated negatively with liver regeneration and increased post-operative complications, rendering THBS1 a predictor of post-hepatectomy liver dysfunction^39^. In line, our data corroborate that the THBS1/ TGF-β axis is a reasonable target of MSC to ameliorate post-hepatectomy multi-organ damage and failure. pBM-MSC do not impact on TGF-β signaling in hepatocytes, but more likely on THBS1-dependent latent TGF-β activation from extracellular matrix-deposited sources. Platelets were identified a major source of THBS1 in the hepatic parenchyma, whereas activated stellate cells and cells residing in the fibrous septae of the pig liver contributed both to parenchymal and septal THBS1 after surgery-induced activation (cf. Figs. 3 and 4). Thrombin is a well-known activator of platelets by stimulating protease-activated receptor 1 signaling and expression of numerous genes including *THBS1*^40^. Indeed, we found increased amounts of platelets in the liver parenchyma after ePHx, whereas at the same time, plasma levels of the thrombin antagonist AT III is low (cf. Fig. 3). AT III is exclusively synthesized by hepatocytes and it is not surprising that levels decrease after resection, due to lower synthetic capacity. AT III is used to predict liver function after resection^41^ and may ameliorate resection-induced liver failure^42^. In this context, we hypothesize that one possible mechanism of MSC is to attenuate THBS1 action by maintaining hepatic AT III production through protection from liver damage. AT III inhibits thrombin through proteolytic degradation, thereby mitigating thrombin-stimulated THBS1 secretion from platelets. In line with our results after ePHx, AT III is low in patients suffering from functional liver impairment in cirrhosis, which seemingly accounts for thromboembolic events such as pulmonary embolism and deep vein thrombosis^43^. Thus, MSC might be a treatment option for this paradox pro-coagulant state observed in patients with cirrhosis and after ePHx. To confirm this hypothesis, MSC need to be established in clinical trials to treat this highly sensitive coagulation/anticoagulation scenario in patients with fatal liver diseases.

In the study presented, the pig may only serve as a surrogate for the human situation. The livers of pigs and humans do not differ in essential functional parameters. Anatomically, there are some differences in the distribution of the liver lobes, but not in the blood supply and the bile duct system. Histologically, the porcine liver features higher septation than the human liver^44^. Due to the good comparability of the two species with respect to liver anatomy and function, the porcine model is often considered an ideal and realistic model for extended liver resections^45^ and liver transplantations^46^. More than that, the use of porcine livers as xenogeneic grafts proves the similarity between these species^47^. Therefore, and considering the fact that THBS1 is a negative predictor of post-hepatectomy liver function in humans^39^, it might be speculated that MSC treatment might also target THBS1 in humans. There is in fact need for action, as, unfortunately, no specific therapeutic options currently exist that can prevent or mitigate acute liver failure after liver resection. The current clinical practice comprises conservative therapeutic measures such as catecholamine therapy, fluid substitution, substitution of albumin, and coagulation factors, binding of potentially toxic substances and support of renal function by hemodialysis. All of these options are not causally effective but are mere supportive measures. The only currently available treatment option in clinical practice is liver transplantation, which is contraindicated in most patients due to the underlying malignancy^48^.

**Fig. 10:**
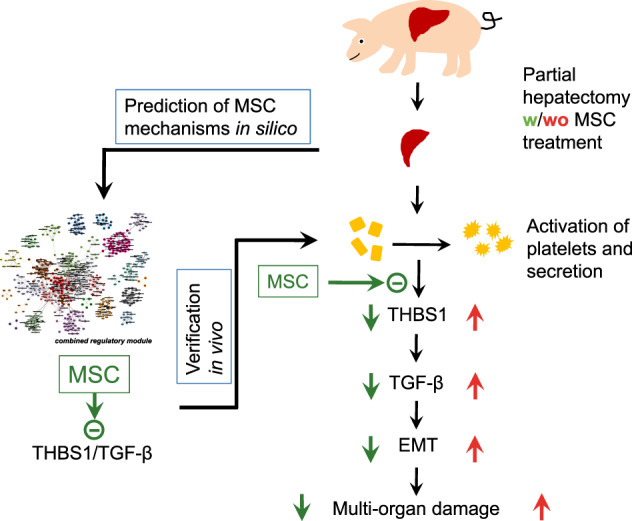
Schematic of how systemically administered MSC may prevent liver surgery-induced damage in different organs. As predicted in silico, MSC inhibit THBS1 secretion from platelets, thus preventing THBS1-mediated TGF-β activation and downstream epithelial-mesenchymal transitions (for details, cf. main text).

We conclude that our results are supporting the hypothesis that extended PHx induces systemic increase in THBS1, consecutively increasing availability of active TGF-β and downstream Smad signaling, both mediators of stress-induced hepatocellular plasticity and EMT. Tightly regulated epithelial cell plasticity is necessary for tissue remodeling during liver regeneration, but in excess due to sustained elevation, THBS1 may be deleterious to the liver and other organs. pBM-MSC inhibit THBS1 secretion, thereby protecting the liver and other organs from damage. Thus, to identify MSC-derived factors targeting THBS1 post surgery might open therapy options to treat PHLF and multi-organ failure (Fig. 10).

## Methods

### Animal trials

Animal experiments were conform to the animal welfare act and approved by the federal state authority of Saxony (file no. TVV39/13). Adult male German landrace pigs were obtained from the farm product company Kitzen (Pegau, Germany) and were housed at the Experimental Centre of the Faculty of Medicine, University of Leipzig, under a 12 h circadian rhythm at 25 °C receiving a standard pig diet for at least 3 days. Animals were starved 24 h before surgery and underwent a veterinary inspection verifying body weight (25–30 kg), temperature (below 40 °C), and general health condition. Animal housing and treatments were in accordance with the Guide for the Care and Use of Laboratory Animals. If not otherwise indicated, nine adult male German landrace pigs were randomized into groups receiving either Ringer solution after ePHx (control), or 1 × 10^8^ hepatocytic differentiated pBM-MSC via central venous transfusion. Sham-treated animals remained without liver resection and central infusion. A minimum of three animals per group was included to enable statistical analyses. However, group size was largely kept at this minimum in order to meet the 3R principles as far as possible.

### Surgical procedure

Seventy percent partial hepatectomy was adapted to Arkadopoulos et al.^49^ and performed essentially as described previously^22^. All animals, including the sham-operated pigs, underwent the same pre-, peri-, and post-surgery procedures as described in more detail in Supplementary Methods (Supplementary Fig. 11), except omission of liver resection in the sham and MSC infusion in the MSC treatment group. Post surgery, the animals were subjected to intensive care monitoring and treatment for 24 h. This time period was chosen according to the procedure described by Arkadopoulos et al.^49^, which turned out to be sufficient to identify surgery-induced early changes indicative for liver damage and acute failure. Following the demands of the animal welfare act, extension of the observation period was restricted to avoid excessive suffer for the animals.

Values of AST, ALT, ammonia, lactate, and the INR were determined in blood samples taken from a horizontal arterial catheter. Depending on the downstream analysis, the various samples were submitted to different procedures immediately after collection. Blood samples collected during the 24 h post-surgery intensive care monitoring were sent directly to the Institute of Laboratory Medicine, Clinical Chemistry and Molecular Diagnostics, University of Leipzig Medical Center, in the vacutainers provided for this specific analysis and immediately processed there accordingly. For the determination of plasma proteins by ELISA, blood was collected in serum vacutainers and immediately centrifuged at 4000 × *g* for 5 min. The supernatant was snap frozen and stored in liquid nitrogen. The ICG-clearance rate was determined before, immediately after (0 h), 12 h, and 24 h post-resection. The ICG-clearance corresponded to the plasma disappearance rate of ICG (PDR_ICG_) and was measured using the LIMON^®^ (Pulsion Medical Systems, Munich, Germany) after a bolus application of 2 mL ICG (ICGPULSION; Pulsion Medical Systems; Rastatt, Germany) via the central venous catheter.

### Human and pBM-MSC isolation and differentiation

Procedures involving hBM-MSC were approved by the Institutional Ethics Review Board Leipzig (file no. 282/11-ek). hBM was obtained from waste material during elective knee or hip joint surgery at the Department of Orthopedics, Trauma and Plastic Surgery, Division of Endoprothetic Joint Surgery/Orthopedics, University of Leipzig Medical Center, after obtaining the patients’ written consent. Isolation, differentiation into the hepatocytic lineage, and characterization of porcine (pBM-MSC) and hBM-MSC has been detailed previously^50,51^. Briefly, MSCs were isolated from the Substantia spongiosa of the Os femoris using collagenase (NB4G, Serva GmbH, Heidelberg, Germany) digestion. The isolated mononuclear cell fraction was seeded on plastic culture dishes and expanded until 90% confluence in stem cell maintenance medium. Thereafter, h/pBM-MSC were differentiated into hepatocyte-like cells using hepatocyte growth medium (HGM) essentially as described^50^. After 14 days, cells were collected in Ringer´s saline and 1 × 10^8^ cells were collected in a transfusion syringe. We used differentiated MSC, because they secreted a significantly broader panel of potentially hepatotropic factors than undifferentiated cells^52^. This is supposed to be advantageous, because during the post-surgery observation period of 24 h as chosen here, it is expected that hepatoprotective mechanisms are not due to MSC tissue integration and substitution. Based on our previous experience from extended PHx in the rat model, rather mechanisms depending on soluble factors were to be expected^9^.

### Gene expression analyses

Gene expression analysis from six lung and six liver samples (each two organ samples from three different animals per group) was performed using Affymetrix Porcine Gene 1.0 ST microarrays at the “KFB - Center of Excellence for Fluorescent Bioanalytics” (Regensburg, Germany; www.kfb-regensburg.de).

#### Total RNA extraction from liver tissue

Approximately 200 mg of porcine liver tissue was homogenized in 700 µL Trizol reagent using Precellys CK14 ceramic beads (VWR, Darmstadt, Germany) (2 cycles of 15 s at 6500 r.p.m.; 10 s break). After 5 min incubation at room temperature, 140 µL of chloroform was added and the samples were again incubated at room temperature for 2 min. Phase separation was achieved by 15 min centrifugation at 12,000 × *g* at 4 °C. An equal amount of 70% ethanol was added to the aqueous supernatant and the mixture was applied to RNeasy Mini spin columns (RNeasy Mini Kit, QIAGEN, Hilden, Germany), followed by an on-column DNase digestion and several wash steps. Finally, total RNA was eluted in 30 μL of nuclease-free water. Purity and integrity of the RNA was assessed on the Agilent 2100 Bioanalyzer with the RNA 6000 Nano LabChip reagent set (Agilent, Palo Alto, CA, USA).

#### Total RNA extraction from lung tissue

Approximately 200 mg of porcine lung tissue was homogenized in 800 µL RLT buffer (Qiagen, Hilden, Germany) using Precellys CK14 ceramic beads (2 cycles of 20 s at 6500 r.p.m.; 15 s break). Next, 1.4 mL of Trizol reagent was added to 150 µL of homogenate. After 5 min at room temperature, 280 µL of chloroform was added and the samples were again incubated at room temperature for 2 min. Phase separation was achieved by 15 min centrifugation at 12,000 × *g* at 4 °C. An equal amount of 70% ethanol was added to the aqueous supernatant and the mixture was applied to RNeasy Microspin columns (RNeasy Micro Kit, QIAGEN, Hilden, Germany), followed by an on-column DNase digestion and several wash steps. Finally, total RNA was eluted in 14 μL of nuclease-free water. Purity and integrity of the RNA was assessed on the Agilent 2100 Bioanalyzer with the RNA 6000 Pico LabChip reagent set (Agilent, Palo Alto, CA, USA).

#### GeneChip microarray assay

Sample preparation for microarray hybridization was carried out as described in the Affymetrix GeneChip WT PLUS Reagent Kit User Manual (Affymetrix, Inc., Santa Clara, CA, USA). In brief, 200 ng of total RNA was used to generate double-stranded cDNA. Then, 15 µg of subsequently synthesized cRNA was purified and reverse transcribed into sense-strand (ss) cDNA, whereas unnatural dUTP residues were incorporated. Purified ss cDNA was fragmented using a combination of uracil DNA glycosylase and apurinic/apyrimidinic endonuclease 1 followed by a terminal labeling with biotin. Next, 3.8 µg fragmented and labeled ss cDNA were hybridized to Affymetrix Porcine Gene 1.0 ST arrays for 16 h at 45 °C in a GeneChip hybridization oven 640. Hybridized arrays were washed and stained in an Affymetrix Fluidics Station FS450 and the fluorescent signals were measured with an Affymetrix GeneChip Scanner 3000 7G. Fluidics and scan functions were controlled by the Affymetrix GeneChip Command Console v4.1.3 software. Sample processing was performed by an Affymetrix Service Provider and Core Facility, “KFB - Center of Excellence for Fluorescent Bioanalytics” (Regensburg, Germany; www.kfb-regensburg.de).

#### Microarray data analysis

Summarized probe set signals in log2 scale were calculated by using the robust multi-array (RMA)^53^ algorithm with the Affymetrix GeneChip Expression Console v1.4 Software. After exporting into Microsoft Excel, average signal values, comparison fold changes, and significance *P*-values were calculated. Probe sets with a fold-change above 2.0-fold and a Student’s *t*-test *P*-value lower than 0.05 were considered as significantly regulated.

#### Gene array data processing

Pre-processing and analysis of gene expression data (available at Gene Expression Omnibus: GSE134970) was performed for each organ separately. Microarray annotation of raw data was achieved using annotation information from Brainarray^54^. Background correction, quantile normalization, and probe summarization was performed using RMA method^55^ as implemented in the affy-package for R^56^. For the lung samples, a batch effect related to the processing of samples was corrected using ComBat^57^ as implemented in the sva package for R^58,59^. Differential gene expression between treatment and control samples was assessed using Limma for R^60^. Based on a *P*-value < 0.05 and a minimal absolute log2 fold-change of at least 0.5, we identified 679 DEGs in the lung and 469 DEGs in the liver.

### Identification of regulatory modules

In summary, regulatory modules were identified using ModuleDiscoverer^23^. The algorithm maps a set of DEGs onto an organism-specific PPIN for the identification of a sub-network (regulatory module), which by statistics is significantly enriched with DEGs.

In order to identify a sub-network, the underlying PPIN is first fragmented into small groups of highly interacting proteins. This is driven by the assumption that within such PPINs, closely connected proteins are involved in similar biological functions^61^. These protein groups are then tested for their significant enrichment with DEGs based on Fisher’s exact test. The union of all significantly enriched protein groups then assembles the regulatory module, which is assumed to include all relevant biological functions and pathways affected by the treatment^61^. The challenge of this strategy lies in the identification of the small groups of proteins. Barrenaes et al.^62^ define them as maximal cliques in the network. A clique is a group of proteins, in which each protein is connected to every other protein within the group. A maximal clique is a clique that cannot be extended by any other protein of the network. However, the identification of all maximal cliques in a network is a non-deterministic polynomial time hard problem, which renders it computationally infeasible for large-scale, whole-genome PPINs. To overcome this problem, the randomization-based heuristic of ModuleDiscoverer enumerates maximal cliques of size three or more in an iterative approach starting from a random seed protein for each iteration. This way, the PPIN’s underlying community structure, i.e., the set of protein groups, is approximated across the full network. ModuleDiscoverer then uses permutation-based *P*-value calculation for the identification of all significantly enriched maximal cliques. In that, for each clique the *P*-value computed based on the set of DEGs is compared to a set of *P*-values based on sets of randomly selected genes of size |DEGs|. These genes are sampled from the statistical background, i.e., the set of genes measured on the microarray. Unification of all significantly enriched maximal cliques then assembles the regulatory module. As ModuleDiscoverer uses a randomization-based approach for the identification of regulatory modules, the stability of the result, i.e., the reproducibility of the identified regulatory module has to be assessed. To this end, ModuleDiscoverer uses bootstrap samples (model-free resampling with replacement) of the set of all enumerated maximal cliques for the identification of additional regulatory modules. The graph-edit distance between every two identified regulatory modules then provides a measure for the average similarity in terms of common edges and nodes in relation to all edges and nodes in the two regulatory modules compared.

### Identification of organ-specific regulatory modules using ModuleDiscoverer

Identification of the regulatory modules for lung and liver using ModuleDiscoverer was performed as follows. The organism-specific PPIN was downloaded from the STRING database version 10^63^. We then removed all edges with an edge-specific confidence score < 0.7 retaining a PPIN composed of 13,507 proteins connected by 184,978 high-confidence relations. Identification of maximal cliques was performed using 2,000,000 iterations starting with one random seed protein per iteration. This identified a total of 1,441,247 maximal cliques containing 133,549 unique maximal cliques. To make use of the permutation-based *P*-value calculation provided by ModuleDiscoverer, we further created 10,000 sets of 679/469 genes (lung/liver) randomly sampled from all 17,299 measured genes on the microarray. Translation of EntrezGene IDs for the set of DEGs and the sets of random genes into EnsemblProtein IDs was then performed using the Ensembl database^64^ via the biomaRt package for R^65^. Based on the set of identified maximal cliques in the PPIN, the set of DEGs and 10,000 sets of random genes, we then identified 334/310 (lung/liver) significantly enriched (*P*-value < 0.01) maximal cliques, which were then unified to assemble the organ-specific regulatory module.

Regulatory modules for lung and liver were identified by ModuleDiscoverer. The lung-specific regulatory module is composed of 358 proteins connected by 1883 relations. Of these 358 proteins, 262 proteins corresponded to genes measured on the microarray including 68 proteins associated to DEGs. The liver-specific regulatory module contained 364 proteins connected by 2587 relations. Two hundred and sixty of all 364 proteins were associated to genes measured on the microarray including 57 DEG-associated proteins. Both of the identified regulatory modules were significantly enriched (*P*-value < 1 × 10^−4^) with proteins associated to DEGs as well as stable (average node/edge stability greater 95%) with respect to regulatory modules identified from 100 additional bootstrap samples of sets of maximal cliques.

### LASSO inferred cluster regulatory network

To identify protein clusters specifically associated with the MSC treatment, we inferred a CRN. To this end, we defined a linear model to estimate the expression value *x* of cluster *i* in sample *m* ($\hat x_i(m)$) based on the sum of the weighted ($\beta _{i,j}$) expression values of all remaining clusters *j* ($x_j(m)$) as well as two weighted variables corresponding to the treatment (1, if sample *m* is treated with MSC; 0 else) and organ (1, if sample *m* is taken from liver; 0 else).

1 $$\begin{array}{l}\hat x_i\left( m \right) = \mathop {\sum }\limits_{j = 1;j \ne i}^C \beta _{i,j} \ast x_j(m) + \beta _{i,C + 1} \ast \left({{{\mathrm{treatment}}}}\left( m \right) + \beta _{i,C + 2} \ast {{{\mathrm{organ}}}}( m )\right)\\ {{{\mathrm{Treatment}}}}\left( m \right) = \left\{ {\begin{array}{ll} 1 & ,{{{{\mathrm{if}}}}\;{{{\mathrm{sample}}}}\;m\;{{{\mathrm{is}}}}\;{{{\mathrm{treatment}}}}} \\ 0 & ,{{{{\mathrm{if}}}}\;{{{\mathrm{sample}}}}\;m\;{{{\mathrm{is}}}}\;{{{\mathrm{control}}}}} \end{array}} \right.\\ {{{\mathrm{Organ}}}}\left( m \right) = \left\{ {\begin{array}{ll} 1 & {,{{{\mathrm{if}}}}\;{{{\mathrm{sample}}}}\;m\;{{{\mathrm{is}}}}\;{{{\mathrm{from}}}}\;{{{\mathrm{liver}}}}} \\ 0 & {,{{{\mathrm{if}}}}\;{{{\mathrm{sample}}}}\;m\;{{{\mathrm{is}}}}\;{{{\mathrm{from}}}}\;{{{\mathrm{lung}}}}} \end{array}} \right.\end{array}$$

We then performed a regression analysis using the LASSO approach as implemented in the glmnet package for R^66^. In this implementation, the LASSO identifies an optimal parameter set $\beta ^ \ast$ that minimizes the difference between the observed ($x_i(m)$) and predicted ($\hat x_i(m)$) expression values based on the mean squared error (Eq. 2). As an additional side constrain, the sum of all absolute parameter values needs to be below some user-defined threshold *λ*, which allows for automated variable selection. Estimation of the optimal *λ* was performed by 12-fold cross-validation.

2 $${{{\mathrm{MSE}}}}\left( \beta \right) = \frac{1}{{M \ast C}}\mathop {\sum }\limits_{m = 1}^M \mathop {\sum }\limits_{i = 1}^C \left( {x_i\left( m \right) - \hat x_i\left( m \right)} \right)^2$$

3 $$\begin{array}{ll}\beta ^\ast =& \mathop {{{{{{{\mathrm{arg}}}}}}\;{{{{{\mathrm{min}}}}}}}}\limits_{\forall \;\beta } {{{\mathrm{MSE}}}}(\beta )\;{{{\mathrm{subject}}}}\;{{{\mathrm{to}}}}\;\mathop {\sum }\limits_{i = 1}^C \left(\left(\mathop {\sum }\limits_{j = 1;j \ne i}^C \left| {\beta _{i,j}} \right| \right)\right.\\ &\left.+ \left| {\beta _{i,C + 1}} \right| + \left| {\beta _{i,C + 2}} \right| \right) \le \lambda\end{array}$$

The process of cross-validation involves random sampling into sets of test and training samples. In order to obtain stable variable selection, we thus performed the cross-validation step 1000 times.

### Identification of upstream regulators

Upstream regulatory genes were identified by IPA (Qiagen, Hilden, Germany) using all genes significantly enriched by MSC treatment compared to the control (*P*-value < 0.05) from the lung and liver. TGF-β1 target genes for both the liver and lung were plotted as regulatory network.

### Cultures of primary pig and mHCs, mouse non-parenchymal liver cells, MDCK II cells, HUVEC, and human THCs

Pig hepatocytes were isolated by a modified two-step collagenase perfusion protocol as described elsewhere^50,67^ and cryopreserved in inactivated fetal calf serum (iFCS) and 7.5% dimethyl sulfoxide in liquid nitrogen until use. For single cultures, hepatocytes were thawed and seeded in minimal essential medium (MEM) containing 0.5 μg/mL insulin (Sigma-Aldrich, Darmstadt, Germany), 50 μg/mL gentamycin (Biochrom GmbH, Berlin, Germany), and 5% iFCS at a density of 300,000 cells/9 cm^2^. Five hours after seeding, the medium was changed to HGM containing 2% iFCS as described^68^. Medium was refreshed after 24 h and cells further grown for 2 days. Before start of the experiments, the medium was sucked off and culture continued for 1 h in serum- and growth factor-free HGM. For co-culture, pHCs were mixed with pBM-MSC (1 : 1) and grown until 80–90% confluence in HGM. TGF-β signaling was studied after stimulation with 1 ng/mL TGF-β (PeproTech GmbH, Hamburg, Germany) for 1 h and pSmad was determined by ELISA or immunocytochemistry. When pig hepatocytes were treated with CM-MSC, the stem cells were cultured for 14–18 days in HGM and CM taken at the end of culture after a medium change 72 h before harvest. CM-MSC was transferred to the hepatocytes and culture continued for 1 h with TGF-β as indicated. pSmad was determined by ELISA as described.

All mouse experiments were approved by the federal state authority of Saxony (reg. no. TVV15/16) and followed all legislation of the animal welfare act. Twelve-week-old, male immune-deficient Pfp/Rag2−/− (C57BL/6N(B6.129S6-Rag2(tm1Fwa)Prf1(tm1Clrk))) mice were housed under standard conditions with a 12 h circadian rhythm at ambient temperature with free access to food (chow diet, V1534, Ssniff, Soest, Germany) and water. mHCs were isolated essentially as described previously^69^. Single cultures were grown in ECM medium (PromoCell GmbH, Heidelberg, Germany) for 48 h before starting the experiments. When hepatocytes were cultured with CM derived from human THCs collected after 10 min of culture (CM-THC), 1 mL of CM-THC medium, or ECM as control medium was added and culture continued for 1 h. THBS1, TGF-β, and pSmad were determined by ELISA.

Non-parenchymal mouse liver cells (NPCs) were isolated from the supernatant of the first centrifugation step in the hepatocyte isolation protocol. The supernatant was re-centrifuged (5 min, 47 × *g*, 4 °C) followed by centrifugation for 10 min at 317 × *g*. The pellet was resuspended in a final volume of 30 mL phosphate-buffered saline (PBS), and centrifuged (10 min, 317 × *g*, 4 °C). This step was repeated until the pellet was void of erythrocytes by visual inspection. The final cell pellet was resuspended in 12 mL ECM containing 1.8 × 10^6^ hepatocytes and plated at 2 mL per well in a six-well plate for co-culture.

To address a direct impact of MSC on TGF-β-induced EMT, we used in vitro co-cultures of hBM-MSC and the epithelial cell line MDCK II (Madin-Darby canine kidney; Sigma-Aldrich as supplied by European Collection of Authenticated Cell Cultures (ECACC 00062107)). MDCK II cells and hBM-MSC (1 : 1) were seeded in alpha MEM (Biochrom GmbH, Berlin, Germany) supplemented with 5% iFCS for 3 days until confluence. After a medium change, cells were stimulated with TGF-β (5 ng/mL) for 20 h where indicated.

Then, 33,000 cells/cm^2^ HUVEC (C-003-5C; Thermo Fischer Scientific GmbH, Dreieich, Germany) were grown in ECM medium (PromoCell GmbH, Heidelberg, Germany) until 80%–90% confluence. For co-culture, HUVECs and hBM-MSCs were seeded at a ratio of 1 : 1 in ECM and grown until 80%–90% confluence for at least 3 days before starting the experiments. Where indicated, HUVECs were treated with CM-MSC collected after 72 h of culture in ECM supplement as described above. Supernatants and cells were collected after 0.25, 2, and 6 h for determination of THBS1 by ELISA or immunocytochemistry, RT-PCR, and western blotting, respectively.

THCs were isolated from plasma donations at the Institute of Transfusion Medicine, University of Leipzig Medical Center, as approved by the Institutional Ethics Review Board Leipzig, after receiving the donors’ written consent. The blood was collected in acid citrate dextrose solution as used for routine blood donations. In order to minimize activation, the platelet concentrates were stored at ambient temperature in storage solution containing 30% plasma with a final citrate concentration of about 3 µM. This procedure still allowed for platelet activation by external stimuli in vitro, since for downstream applications the platelets were suspended or diluted in non-citrate containing media. Immediately after the isolation procedure, THC suspensions (11–14 × 10^8^ THC) were centrifuged (800 × *g*, 5 min, 24 °C) and resuspended 1 : 1 (wt/v) in ECM medium (PromoCell GmbH, Heidelberg, Germany), or in CM (CM-MSC) from hBM-derived hepatocyte-differentiated MSC (hBM-MSC) collected after 72 h of culture in ECM. After 10 min of incubation at 37 °C with or without 10 U/mL thrombin^70^, the medium was collected, centrifuged (5 min, 1000 × *g*, 24 °C), and used for cultures with mHCs as described above, or determination of THBS1 by ELISA.

### Immunohistochemical detection of E-cadherin, N-cadherin, ZO-1, HS, CD31, CD42b, α-SMA, THBS1, TGF-β, and Smad 2/3

Paraffin-embedded tissue slices (1.5 µm) were incubated with TRIS-buffer (10 mM Tris-HCl, 1 mM EDTA, pH 9.0) and blocking solution (5% goat serum) was added thereafter for 20 min followed by blocking for 60 min in blocking solution made of 5% bovine serum albumin (BSA) and 0.5% Tween 20. Then, slices were incubated with the primary anti-E-cadherin, anti-N-cadherin, anti-ZO-1, anti-HS, anti-CD31, anti-CD42b, or the anti-α-SMA antibody (Supplementary Table 1) overnight at 4 °C. Subsequent to three washing steps with PBS, the secondary antibodies labeled with Cy3 or AlexaFluor488 (Supplementary Table 1) were applied for 70 min at room temperature. After another washing with PBS, slices were counterstained with DAPI solution (Roth GmbH, Karlsruhe, Germany) and embedded in glycerol solution (50%; Roth GmbH, Karlsruhe, Germany) for microscopic analysis. For detection of THBS1 or TGF-β, tissue slices were incubated in citrate buffer (100 mM citric acid, 100 mM sodium citrate pH 6.0). After treatment with H_2_O_2_ (3%), BSA (5%), and Avidin/Biotin (Vector- Kit SP-2001, BIOZOL GmbH, Eching, Germany) blocking solution, slices were incubated with the anti-THBS1 or the anti-TGF-β antibody overnight at 4 °C (Supplementary Table 1). In addition to three washing steps with PBS, the biotin-labeled secondary antibody (Supplementary Table 1) was incubated with the tissue slices for 60 min at room temperature. Subsequent to three washing steps with PBS, ABC-reagent (Vector- Kit PK-6100, Vectorlabs, Burlingame, CA, USA) was applied for 30 min. After another PBS washing step, DAB-solution (Pierce™ DAB Substrate Kit, Thermo Fischer Scientific GmbH, Dreieich, Germany) was used for color development of THBS1 and NovaRed (Vector- Kit SK-4800, Vectorlabs, Burlingame, CA, USA) for TGF-β. Slices were counterstained with nuclear fast red solution (Roth GmbH, Karlsruhe, Germany) for THBS1 and Mayer’s hemalum solution (Merck, Darmstadt, Germany) for TGF-β, and embedded in Entellan (Merck GmbH, Darmstadt, Germany).

For color development of Smad 2/3, a horseradish peroxidase (HRP)-linked secondary antibody was used instead of avidin/biotin in combination with Histogreen (Linaris- Kit- E-109, Dossenheim, Germany).

Slices were counterstained with nuclear fast red solution (Roth GmbH, Karlsruhe, Germany) and embedded in Entellan (Merck GmbH, Darmstadt, Germany).

### Immunocytochemistry

For staining of Smad 2/3 and Phalloidin in co-cultures of pHCs and pBM-MSC, cells were seeded on collagen-coated coverslips. Coverslips were washed twice in PBS and fixed with formalin (3.7%) for 15 min. In addition to two further PBS washing steps, blocking solutions were added (5% goat serum for 20 min and 5% BSA for another 60 min). The anti-Smad 2/3 antibody (Supplementary Table 1) was administered overnight at 4 °C. After incubation with the secondary antibody AlexaFluor488 (Supplementary Table 1) for 50 min, the cytoskeleton was visualized with Phalloidin 568 (1 : 500) (Thermo Fisher Scientific GmbH, Dreieich, Germany) for 50 min. Following another washing with PBS, cells were counterstained with DAPI solution (Roth GmbH, Karlsruhe, Germany) and embedded in glycerol (50%; Roth GmbH, Karlsruhe, Germany) for microscopic analysis.

For staining of ZO-1 in co-cultures of MDCK II cells and hBM-MSC, cells were seeded on fibronectin-coated coverslips. After two washings with PBS, cells were fixed with 3.7% formalin, again washed twice with PBS, and further incubated with 5% goat serum in PBS for 25 min at room temperature, followed by 60 min incubation in BSA blocking solution (5% BSA and 0.5% Tween 20 in PBS). The primary anti-ZO-1 antibody (Supplementary Table 1) in 1% BSA in PBS was added overnight at 4 °C, followed by three washings with PBS at room temperature for 10 min each. The secondary antibody AlexaFluor488 (Supplementary Table 1) in 0.5% BSA in PBS was added for 60 min followed by three washings with PBS at room temperature. Coverslips were embedded in Prolong with DAPI (Thermo Fisher Scientific GmbH, Dreieich, Germany) for microscopic analysis. For quantification of cell contacts, ZO-1-stained membranes on fluorescence microscopy pictures were manually delineated and total lengths quantified using ImageJ 1.46 (National Institutes of Health, Bethesda, MD, USA).

To stain HUVEC for THBS1, cells were treated as described before for ZO-1. After blocking, the primary anti-THBS1 antibody (Supplementary Table 1) in 1% BSA in PBS was added overnight at 4 °C, followed by three washings with PBS at room temperature for 5 min each. The secondary Cy3-labeled goat anti-mouse antibody (Supplementary Table 1) in 0.5% BSA in PBS was added for 45 min at 37 °C followed by three washings with PBS at room temperature. Phalloidin iFluor 488 (Supplementary Table 1) in 0.5% BSA in PBS was added for 45 min at 37 °C followed by three washings with PBS and embedding in in Prolong with DAPI (Thermo Fisher Scientific GmbH, Dreieich, Germany).

### Western blot analyses

Western blottings after electrophoretic separation under denaturing conditions were performed according to standard protocols. In brief, liver tissue was homogenized in RIPA buffer (20 mM Tris-HCl (pH 7.5), 150 mM NaCl, 1 mM EDTA, 1 mM EGTA, 1% Triton X-100, 1% Na-deoxycholate) supplemented with protease- and phosphatase inhibitor tablets (Roche, Basel, Switzerland). Twenty micrograms of protein lysates were separated on 10% SDS-polyacrylamide gels and transferred onto nitrocellulose membranes according to standard protocols. Primary antibodies were applied overnight at 4 °C and proteins detected by HRP-linked secondary antibodies (Supplementary Table 1).

### ELISA assays

#### TGF-β1

Plasma and culture medium levels were determined in duplicates by using the porcine TGF-β1-ELISA (DlDevelop, Kelowna, BC, Canada) according to the provider’s manual. Then, 100 µL of porcine blood plasma or hepatocyte culture medium were incubated for 120 min at 37 °C in the ELISA microtiter plate. Following three washing steps (300 µL 1× wash buffer), 100 µL detection reagent A were added for 60 min at 37 °C. After another four washing steps (300 µL washing buffer), the chromogen solution (90 µL) was applied for at least 15–25 min at 37 °C. After incubation with 50 µL stop solution, chromogen formation was measured (450 nm) using the GloMax®-Multi Detection System (Promega, Mannheim, Germany). Standard dilutions of TGF-β1 in the range of 0–1000 pg/mL were treated analogously to the samples and used for the calculation of TGF-β1 concentrations.

#### Antithrombin III

Plasma levels were determined in duplicates by using the porcine Antithrombin-ELISA Kit (MyBioSource, San Diego, CA, USA) according to the instructions. Fifty microliters of plasma and 100 µL of HRP-conjugate were incubated in the ELISA microtiter plate for 60 min at 37 °C. After four washings (250 µL washing buffer each), 50 µL each of chromogen solution A and B were added and incubated for 15 min at 37 °C in the dark. Reaction was stopped by adding 50 µL stop solution and chromogen formation was measured at 450 nm using the GloMax®-Multi Detection System (Promega, Mannheim, Germany). Values shown in Fig. 3b were corrected for the amount of AT III at the zero time point.

#### Thrombospondin-1

Levels in porcine plasma during 24 h post surgery and in HUVEC and THC culture supernatants were determined in duplicates by the human THBS1-ELISA (human Quantikine®ELISA R&D Systems, Abingdon, UK) according to the instructions. In brief, 100 µL Assay-Diluent and 50 µL plasma (1 : 20 in Calibrator Diluent RD5-33) or supernatant (1 : 2 in Calibrator Diluent RD5-33) were filled into the wells of the ELISA plate and incubated for 2 h at room temperature. After four washing steps, 200 µL THBS1-conjugate solution was added to each well for 2 h. After another four washing steps, 200 µL substrate solution was added for 30 min. Reaction was stopped by adding 50 µL stop solution and chromogen formation was measured at 450 nm using the GloMax®-Multi Detection System (Promega, Mannheim, Germany).

#### Phospho-Smad 2/3

Phospho-Smad 2/3 was determined using the PathScan® Phospho-Smad 2 (Ser465/467)/Smad 3 (Ser423/425) Sandwich ELISA (Cell Signaling Technology, Frankfurt, Germany) according to the manufacturer’s instructions. In brief, 50 mg of porcine liver tissue or cell pellets (mouse or pig) of one well of a six-well plate were lysed in 500 µL of 1× lysis buffer. Lysates were diluted 1 : 2 by using sample-diluent. Next, 100 µL of diluted samples were incubated overnight at 4 °C in the microwells. After four washing steps (200 µL of 1× wash buffer), 100 µL of detection antibody were added to the wells for 1 h at 37 °C. After another four washing steps, 100 µL of HRP-linked secondary antibody were added for 30 min at 37 °C. Following four washings, 100 µL 3,3', 5,5''-tetramethylbenzidine substrate were added for 10 min. After incubation with 100 µL stop solution, absorbance was measured at 450 nm using the GloMax®-Multi Detection System (Promega, Mannheim, Germany). Relative absorbance was normalized to 100 µg of total protein where indicated. Each sample was run in duplicate in the assay.

#### PF4

PF4 in pig plasma and liver tissue was determined with the PF4 ELISA kit obtained from ELK Biotechnology (Hölzel Diagnostika Handels GmbH, Köln, Germany) using the manufacturer´s instructions. Next, 100 mg of liver tissue were homogenized in 1 mL of ice-cold PBS and centrifuged at 10,000 × *g* for 5 min. One hundred microliters of the supernatant or 100 µL porcine plasma were incubated on the ELISA plates at 37 °C for 2 h, while shaking. Supernatants were sucked off and 100 µL of the diluted biotin-antibody conjugate were added and incubation continued for 1 h. Following three washing steps for 2 min each with wash buffer, 100 µL of HRP-Avidin conjugate were added and incubation continued for 1 h. Following another three washings, 90 µL of the substrate solution were added and incubation continued in the dark for 15–30 min. After adding 50 µL stop solution, absorbance was measured at 450 nm using using the GloMax®-Multi Detection System (Promega, Mannheim, Germany).

#### PDGF-AB

PDGF-AB in pig plasma and liver tissue was determined with the PDGF-AB ELISA kit obtained from CUSABIO (Hölzel Diagnostika Handels GmbH, Köln, Germany) using the manufacturer’s instructions. Liver samples were prepared and the procedure continued as described above for PF4.

#### THBS1

THBS1 at 24 h after ePHx was determined using the ELISA Kit for THBS1 obtained from Cloud-Clone Corp. (Katy, TX, USA) according to the manufacturer’s manual. Liver tissue samples were prepared as described above for PF4 and 100 µL of tissue extract or 100 µL of plasma were incubated on the ELISA plates at 37 °C for 2 h, while shaking. Supernatants were sucked off and 100 µL of the diluted detection reagent A were added and incubation continued for 1 h. Following three washing steps for 2 min each with wash buffer, 100 µL of diluted detection reagent B were added and incubation continued for 30 min. Following another three washings, 90 µL of the substrate solution were added and incubation continued in the dark for 10–20 min. After adding 50 µL stop solution, absorbance was measured at 450 nm using the GloMax®-Multi Detection System (Promega, Mannheim).

### Semi-quantitative RT-PCR

Total RNA was extracted using a standard Trizol protocol. The cDNA synthesis was performed with the Maxima-H-Minus-First-Strand kit (Thermo Fisher, Dreieich, Germany). Then, 100 ng of cDNA were amplified and PCR products analyzed after electrophoretic separation by quantifying the relative intensity of specific bands using ImageJ (v1.42, National Institutes of Health, Bethesda, MA, USA) where indicated. A list of primers is presented in Supplementary Table 2.

### Cytokine array analysis

Cytokines in supernatants of THC were detected using 100 µl of medium according to the manufacturer’s procedure using the Proteome Profiler Human XL Cytokine Array Kit (R&D Systems, Abingdon, UK). Next, 100 µL of CM-THC either with or without CM-MSC were applied according to the manufacturer’s instructions. The array detected proteins as listed in Supplementary Table 3. Labeled proteins were visualized with the Micro Chemi 4.2 using the gel capture software (Biostep, Burkhardtsdorf, Germany) by taking serial pictures with an exposure time of 9 min. Signal intensities were quantified using the ImageJ 1.46 software (NIH, Bethesda, MD, USA) and background correction by the negative controls. Mean pixel density of reference spots was set to 100, to which all other values are relative. Proteins below an abundance of 5 were ignored. For better clarity, Fig. 9b does only comprise cytokines known to be contained in THC α-granules.

### Statistics

If not otherwise indicated in the figure legends, the SPSS software (v24, IBM, Ehningen, Germany) was used for statistical analyses. To verify statistical standard distribution, the *t*-test or the one-way analysis of variance test were applied. Values were considered different at *P* ≤ 0.05.

### Reporting summary

Further information on research design is available in the Nature Research Reporting Summary linked to this article.

## Supplementary information

Reporting Summary

Supplementary information

Supplementary Data 1

## Data Availability

The authors declare that the data supporting the findings of this study are available within the paper and its Supplementary Information files. Otherwise, data generated and analyzed during the current study are available from the corresponding author on reasonable request. Gene expression data that support the findings of this study have been deposited in Gene Expression Omnibus (GEO) with the accession code GSE134970.

## Code availability

For microarray data analyses and subsequent module detection, only publicly available programs were used and applied as outlined in the “Methods” section and/or the supplement.

## References

[1] Uccelli A, de Rosbo NK (2015). The immunomodulatory function of mesenchymal stem cells: mode of action and pathways. Ann. N. Y. Acad. Sci..

[2] Prockop DJ (2016). Inflammation, fibrosis, and modulation of the process by mesenchymal stem/stromal cells. Matrix Biol..

[3] Ezquer M, Ezquer F, Ricca M, Allers C, Conget P (2011). Intravenous administration of multipotent stromal cells prevents the onset of non-alcoholic steatohepatitis in obese mice with metabolic syndrome. J. Hepatol..

[4] Winkler S (2014). Human mesenchymal stem cells towards non-alcoholic steatohepatitis in an immunodeficient mouse model. Exp. Cell Res..

[5] Stock P, Bruckner S, Winkler S, Dollinger MM, Christ B (2014). Human bone marrow mesenchymal stem cell-derived hepatocytes improve the mouse liver after acute acetaminophen intoxication by preventing progress of injury. Int. J. Mol. Sci..

[6] Liu Z (2014). Human umbilical cord mesenchymal stromal cells rescue mice from acetaminophen-induced acute liver failure. Cytotherapy.

[7] Shi D (2017). Quantitative evaluation of human bone mesenchymal stem cells rescuing fulminant hepatic failure in pigs. Gut.

[8] Baligar P (2017). Bone marrow stem cell therapy partially ameliorates pathological consequences in livers of mice expressing mutant human alpha1-antitrypsin. Hepatology.

[9] Tautenhahn HM (2016). Attenuation of postoperative acute liver failure by mesenchymal stem cell treatment due to metabolic implications. Ann. Surg..

[10] Apostolou KG (2018). Undifferentiated adipose tissue stem cell transplantation promotes hepatic regeneration, ameliorates histopathologic damage of the liver, and upregulates the expression of liver regeneration- and liver-specific genes in a rat model of partial hepatectomy. Stem Cells Int..

[11] Guglielmi A, Ruzzenente A, Conci S, Valdegamberi A, Iacono C (2012). How much remnant is enough in liver resection?. Digestive Surg..

[12] Schindl MJ (2005). The value of residual liver volume as a predictor of hepatic dysfunction and infection after major liver resection. Gut.

[13] Lafaro K (2015). Defining post hepatectomy liver insufficiency: where do we stand?. J. Gastrointest. Surg..

[14] Balzan S (2005). The “50-50 criteria” on postoperative day 5: an accurate predictor of liver failure and death after hepatectomy. Ann. Surg..

[15] Chin KM (2020). Early prediction of post-hepatectomy liver failure in patients undergoing major hepatectomy using a PHLF prognostic nomogram. World J. Surg..

[16] Mahmud N (2021). Novel risk prediction models for post-operative mortality in patients with cirrhosis. Hepatology.

[17] Starlinger P (2016). The profile of platelet alpha-granule released molecules affects postoperative liver regeneration. Hepatology.

[18] Kuroki H (2015). Effect of LSKL peptide on thrombospondin 1-mediated transforming growth factor beta signal activation and liver regeneration after hepatectomy in an experimental model. Br. J. Surg..

[19] Hayashi H, Sakai K, Baba H, Sakai T (2012). Thrombospondin-1 is a novel negative regulator of liver regeneration after partial hepatectomy through transforming growth factor-beta1 activation in mice. Hepatology.

[20] Braun L (1988). Transforming growth factor beta mRNA increases during liver regeneration: a possible paracrine mechanism of growth regulation. Proc. Natl Acad. Sci. USA.

[21] Choi SS, Diehl AM (2009). Epithelial-to-mesenchymal transitions in the liver. Hepatology.

[22] Tautenhahn HM (2017). Mesenchymal stem cells correct haemodynamic dysfunction associated with liver injury after extended resection in a pig model. Sci. Rep..

[23] Vlaic S (2018). ModuleDiscoverer: identification of regulatory modules in protein-protein interaction networks. Sci. Rep..

[24] Yip AM, Horvath S (2007). Gene network interconnectedness and the generalized topological overlap measure. BMC Bioinformtics..

[25] Adams JC (1997). Thrombospondin-1. Int. J. Biochem. Cell Biol..

[26] Sipes JM, Murphy-Ullrich JE, Roberts DD (2018). Thrombospondins: purification of human platelet thrombospondin-1. Methods Cell Biol..

[27] Schultz-Cherry S, Murphy-Ullrich JE (1993). Thrombospondin causes activation of latent transforming growth factor-beta secreted by endothelial cells by a novel mechanism. J. Cell Biol..

[28] Resovi A, Pinessi D, Chiorino G, Taraboletti G (2014). Current understanding of the thrombospondin-1 interactome. Matrix Biol..

[29] Willis BC, Borok Z (2007). TGF-beta-induced EMT: mechanisms and implications for fibrotic lung disease. Am. J. Physiol. Lung Cell. Mol. Physiol..

[30] Kim SH, Turnbull J, Guimond S (2011). Extracellular matrix and cell signalling: the dynamic cooperation of integrin, proteoglycan and growth factor receptor. J. Endocrinol..

[31] Yadav S, Storrie B (2017). The cellular basis of platelet secretion: emerging structure/function relationships. Platelets.

[32] Gomes N, Legrand C, Fauvel-Lafeve F (2005). Shear stress induced release of von Willebrand factor and thrombospondin-1 in HUVEC extracellular matrix enhances breast tumour cell adhesion. Clin. Exp. Metastasis.

[33] Oh SH, Swiderska-Syn M, Jewell ML, Premont RT, Diehl AM (2018). Liver regeneration requires Yap1-TGFbeta-dependent epithelial-mesenchymal transition in hepatocytes. J. Hepatol..

[34] Manmadhan S, Ehmer U (2019). Hippo signaling in the liver - a long and ever-expanding story. Front. Cell Dev. Biol..

[35] Dooley S (2008). Hepatocyte-specific Smad7 expression attenuates TGF-beta-mediated fibrogenesis and protects against liver damage. Gastroenterology.

[36] Murphy-Ullrich JE, Poczatek M (2000). Activation of latent TGF-beta by thrombospondin-1: mechanisms and physiology. Cytokine Growth Factor Rev..

[37] Fan XL, Zhang Y, Li X, Fu QL (2020). Mechanisms underlying the protective effects of mesenchymal stem cell-based therapy. Cell. Mol. life Sci..

[38] Eigenthaler M, Nolte C, Halbrugge M, Walter U (1992). Concentration and regulation of cyclic nucleotides, cyclic-nucleotide-dependent protein kinases and one of their major substrates in human platelets. Estimating the rate of cAMP-regulated and cGMP-regulated protein phosphorylation in intact cells. Eur. J. Biochem..

[39] Starlinger P (2015). Plasma thrombospondin 1 as a predictor of postoperative liver dysfunction. Br. J. Surg..

[40] Baenziger NL, Brodie GN, Majerus PW (1972). Isolation and properties of a thrombin-sensitive protein of human platelets. J. Biol. Chem..

[41] Pereyra D (2017). Early prediction of postoperative liver dysfunction and clinical outcome using antithrombin III-activity. PLoS ONE.

[42] Kuroda S (2015). Administration of antithrombin III attenuates posthepatectomy liver failure in hepatocellular carcinoma. Digestive Surg..

[43] Dhar A, Mullish BH, Thursz MR (2017). Anticoagulation in chronic liver disease. J. Hepatol..

[44] Court FG (2003). Segmental nature of the porcine liver and its potential as a model for experimental partial hepatectomy. Br. J. Surg..

[45] Golriz M (2017). Establishing a porcine model of small for size syndrome following liver resection. Can. J. Gastroenterol. Hepatol..

[46] Esmaeilzadeh M (2012). Technical guidelines for porcine liver allo-transplantation: a review of literature. Ann. Transplant..

[47] Lu T, Yang B, Wang R, Qin C (2019). Xenotransplantation: current status in preclinical research. Front. Immunol..

[48] Stravitz RT, Lee WM (2019). Acute liver failure. Lancet.

[49] Arkadopoulos N (2011). Development of a porcine model of post-hepatectomy liver failure. J. Surg. Res..

[50] Bruckner S (2014). A fat option for the pig: hepatocytic differentiated mesenchymal stem cells for translational research. Exp. Cell Res..

[51] Stock P (2010). The generation of hepatocytes from mesenchymal stem cells and engraftment into murine liver. Nat. Protoc..

[52] Winkler S (2016). Identification of pathways in liver repair potentially targeted by secretory proteins from human mesenchymal stem cells. Int. J. Mol. Sci..

[53] Irizarry RA (2003). Exploration, normalization, and summaries of high density oligonucleotide array probe level data. Biostatistics.

[54] Dai M (2005). Evolving gene/transcript definitions significantly alter the interpretation of GeneChip data. Nucleic Acids Res..

[55] Irizarry RA (2003). Summaries of Affymetrix GeneChip probe level data. Nucleic acids Res..

[56] Gautier L, Cope L, Bolstad BM, Irizarry RA (2004). affy−Analysis of Affymetrix GeneChip data at the probe level. Bioinformatics.

[57] Johnson WE, Li C, Rabinovic A (2007). Adjusting batch effects in microarray expression data using empirical Bayes methods. Biostatistics.

[58] Leek JT, Johnson WE, Parker HS, Jaffe AE, Storey JD (2012). The sva package for removing batch effects and other unwanted variation in high-throughput experiments. Bioinformatics.

[59] Leek, J. T. et al. sva: Surrogate Variable Analysis, R package version 3.18.0. (2015).

[60] Ritchie ME (2015). limma powers differential expression analyses for RNA-sequencing and microarray studies. Nucleic Acids Res..

[61] Barabasi AL, Gulbahce N, Loscalzo J (2011). Network medicine: a network-based approach to human disease. Nat. Rev. Genet..

[62] Barrenas F (2012). Highly interconnected genes in disease-specific networks are enriched for disease-associated polymorphisms. Genome Biol..

[63] Szklarczyk D (2017). The STRING database in 2017: quality-controlled protein-protein association networks, made broadly accessible. Nucleic Acids Res..

[64] Aken, B. L., et al. The Ensembl gene annotation system. *Database* **2016**, baw093 (2016). 10.1093/database/baw093 PMC4919035 27337980

[65] Durinck S, Spellman PT, Birney E, Huber W (2009). Mapping identifiers for the integration of genomic datasets with the R/Bioconductor package biomaRt. Nat. Protoc..

[66] Friedman J, Hastie T, Tibshirani R (2010). Regularization paths for generalized linear models via coordinate descent. J. Stat. Softw..

[67] Seglen PO (1976). Preparation of isolated rat liver cells. Methods cell Biol..

[68] Schneider C, Aurich H, Wenkel R, Christ B (2006). Propagation and functional characterization of serum-free cultured porcine hepatocytes for downstream applications. Cell tissue Res..

[69] Winkler S (2019). Immune-deficient Pfp/Rag2(-/-) mice featured higher adipose tissue mass and liver lipid accumulation with growing age than wildtype C57BL/6N mice. Cells.

[70] Joshi N (2015). Coagulation-driven platelet activation reduces cholestatic liver injury and fibrosis in mice. J. thrombosis Haemost..

