# Supplementary material for: Mesenchymal stromal cells mitigate liver damage after extended resection in the pig by modulating thrombospondin-1/TGF-β
Source: NPJ Regen Med. 2021 Dec 3;6:84. doi: 10.1038/s41536-021-00194-4 (PMC8642541; doi:10.1038/s41536-021-00194-4)
Supplement: Supplementary file 1 — Reporting Summary [file 41536_2021_194_MOESM1_ESM.pdf]

nature portfolio

Corresponding author(s): Bruno Christ
Last updated by author(s): Aug 31, 2021

Reporting Summary

Nature Portfolio wishes to improve the reproducibility of the work that we publish. This form provides structure for consistency and transparency in reporting. For further information on Nature Portfolio policies, see our Editorial Policies and the Editorial Policy Checklist.

Statistics

For all statistical analyses, confirm that the following items are present in the figure legend, table legend, main text, or Methods section.

n/a	Confirmed	
☐	☒	The exact sample size (n) for each experimental group/condition, given as a discrete number and unit of measurement
☐	☒	A statement on whether measurements were taken from distinct samples or whether the same sample was measured repeatedly
☐	☒	The statistical test(s) used AND whether they are one- or two-sided<br>*Only common tests should be described solely by name; describe more complex techniques in the Methods section.*
☒	☐	A description of all covariates tested
☐	☒	A description of any assumptions or corrections, such as tests of normality and adjustment for multiple comparisons
☐	☒	A full description of the statistical parameters including central tendency (e.g. means) or other basic estimates (e.g. regression coefficient) AND variation (e.g. standard deviation) or associated estimates of uncertainty (e.g. confidence intervals)
☒	☐	For null hypothesis testing, the test statistic (e.g. F, t, r) with confidence intervals, effect sizes, degrees of freedom and P value noted<br>*Give P values as exact values whenever suitable.*
☒	☐	For Bayesian analysis, information on the choice of priors and Markov chain Monte Carlo settings
☒	☐	For hierarchical and complex designs, identification of the appropriate level for tests and full reporting of outcomes
☒	☐	Estimates of effect sizes (e.g. Cohen's d, Pearson's r), indicating how they were calculated

Our web collection on statistics for biologists contains articles on many of the points above.

Software and code

Policy information about availability of computer code

Data collection	Gene expression analysis: Affymetrix Fluidics Station FS450, and the fluorescent signals were measured with an Affymetrix GeneChip Scanner 3000 7G. Fluidics and scan functions were controlled by the Affymetrix GeneChip Command Console v4.1.3 software.<br>Immunofluorescence: Zeiss Axio Observer.Z1 with integrated software<br>Immunohistochemistry: Zeiss Axio Imager.A1 with integrated software<br>Cytokine arrays: Micro Chemi 4.2 using the gel capture software<br>ELISA: GloMax®-Multi Detection System
Data analysis	Regulatory modules for lung and liver were identified by ModuleDiscoverer<br>Upstream regulatory genes were identified by Ingenuity Pathway Analysis (IPA; Qiagen, Hilden, Germany)

For manuscripts utilizing custom algorithms or software that are central to the research but not yet described in published literature, software must be made available to editors and reviewers. We strongly encourage code deposition in a community repository (e.g. GitHub). See the Nature Portfolio guidelines for submitting code & software for further information.

Data

Policy information about availability of data

All manuscripts must include a data availability statement. This statement should provide the following information, where applicable:

- Accession codes, unique identifiers, or web links for publicly available datasets
- A description of any restrictions on data availability
- For clinical datasets or third party data, please ensure that the statement adheres to our policy

For microarray data analyses and subsequent module detection only publicly available programs were used and applied as outlined in the methods section.

Field-specific reporting

Please select the one below that is the best fit for your research. If you are not sure, read the appropriate sections before making your selection.

☒ Life sciences ☐ Behavioural & social sciences ☐ Ecological, evolutionary & environmental sciences

For a reference copy of the document with all sections, see nature.com/documents/nr-reporting-summary-flat.pdf

Life sciences study design

All studies must disclose on these points even when the disclosure is negative.

Sample size	A minimum of three pigs per group was included to enable statistical analyses. However, group size was largely kept at this minimum in order to meet the 3R principles as far as possible. Cell culture experiments were repeated at least three times with different batches of cell lines or primary cells.
Data exclusions	No data were excluded from the analysis
Replication	Animal experiments involving pigs may not be repeated because of reasons of animal protection (3R principles). Cell culture experiments were repeated at least 3-times yielding comparable results.
Randomization	Pigs were randomized to the 3 experimental groups: sham, w/o MSC treatment, w MSC treatment.
Blinding	Since the experimentalists were the persons involved both in animal trials and data analysis, no blinding was reasonable and possible.

Reporting for specific materials, systems and methods

We require information from authors about some types of materials, experimental systems and methods used in many studies. Here, indicate whether each material, system or method listed is relevant to your study. If you are not sure if a list item applies to your research, read the appropriate section before selecting a response.

Materials & experimental systems

n/a	Involved in the study	
☐	☒	Antibodies
☐	☒	Eukaryotic cell lines
☒	☐	Palaeontology and archaeology
☐	☒	Animals and other organisms
☒	☐	Human research participants
☒	☐	Clinical data
☒	☐	Dual use research of concern

Methods

n/a	Involved in the study	
☒	☐	ChIP-seq
☒	☐	Flow cytometry
☒	☐	MRI-based neuroimaging

Antibodies

Antibodies used	We used 33 different antibodies in this study for immunohisto- and cytochemistry as well as for Western blotting. Their source, dilution and specificities are listed in Supplementary Table 1 of the manuscript. Technically, it is not possible to list all the antibodies here in this field.
Validation	The specificity of each antibody was confirmed by running negative controls omitting the primary antibodies. Specific staining was verified by the specific morphological pattern of staining (e.g. E-cadherin in periportal hepatocytes, ZO-1 for bile canaliculi between adjacent hepatocytes, heparan sulfate at the cell mebrane, nuclear pSMAD etc.).

Eukaryotic cell lines

Policy information about cell lines

Cell line source(s)	C-003-5C; Human Umbilical Vein Endothelial Cells, Thermo Fischer Scientific GmbH<br>MDCK II (Madin-Darby canine kidney; Sigma-Aldrich) as supplied by European Collection of Authenticated Cell Cultures (ECACC 00062107))
Authentication	For HUVEC Thermo states, that each lot of cells is tested immunocytochemically for the presence of vWF and CD31 and abesence of alpha-actin.<br>MDCK: European Collection of Authenticated Cell Cultures (ECACC 00062107))
Mycoplasma contamination	HUVEC and MDCK were tested negatively by the provider.

Commonly misidentified lines (See ICLAC register)	n/a

Animals and other organisms

Policy information about studies involving animals; ARRIVE guidelines recommended for reporting animal research

Laboratory animals	Adult male German landrace pigs (25-30 kg) were obtained from the farm product company Kitzen (Pegau, Germany). 12 weeks old, male immune-deficient Pfp/Rag2-/- (C57BL/6N(B6.129S6-Rag2(tm1Fwa)Prf1(tm1Clrk))) mice were initially from Taconic, Ejby (DK) and then housed under standard conditions at the Experimental Centre of the Faculty of Medicine, University of Leipzig
Wild animals	n/a
Field-collected samples	n/a
Ethics oversight	Pig experiments were conform to the animal welfare act and approved by the federal state authority of Saxony (file no. TVV39/13). All mouse experiments were approved by the federal state authority of Saxony (reg.no. TVV15/16) and followed all legislation of the animal welfare act.

Note that full information on the approval of the study protocol must also be provided in the manuscript.
