# Supplementary material for: Mesenchymal stromal cells mitigate liver damage after extended resection in the pig by modulating thrombospondin-1/TGF-β
Source: NPJ Regen Med. 2021 Dec 3;6:84. doi: 10.1038/s41536-021-00194-4 (PMC8642541; doi:10.1038/s41536-021-00194-4)
Supplement: Supplementary file 2 — Supplementary information [file 41536_2021_194_MOESM2_ESM.pdf]

<u>Title:</u> Mesenchymal stromal cells mitigate liver damage after extended liver resection in the pig by modulating thrombospondin-1/TGF-β

<u>Authors:</u>
Sandra Nickel, Sebastian Vlaic, Madlen Christ, Kristin Schubert, Reinhard Henschler, Franziska Tautenhahn, Caroline Burger, Hagen Kühne, Silvio Erler, Andreas Roth, Christiane Wild, Janine Brach, Seddik Hammad, Claudia Gittel, Manja Baunack, Undine Lange, Johannes Broschewitz, Peggy Stock, Isabella Metelmann, Michael Bartels, Uta Pietsch, Sebastian Krämer, Uwe Eichfeld, Martin von Bergen, Steven Dooley, Hans-Michael Tautenhahn, Bruno Christ

Supplementary Results

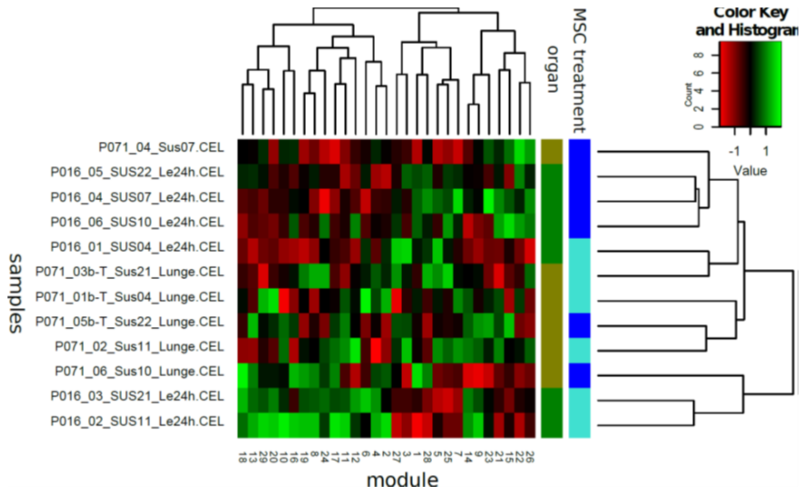

Supplementary Figure 1. EigenGenes For SubModules Heatmap Processed. A heatmap of the scaled expression values for each module derived from the topological clustering based on the general topological overlap measure showed that the samples clustered mostly by treatment, not by organ.

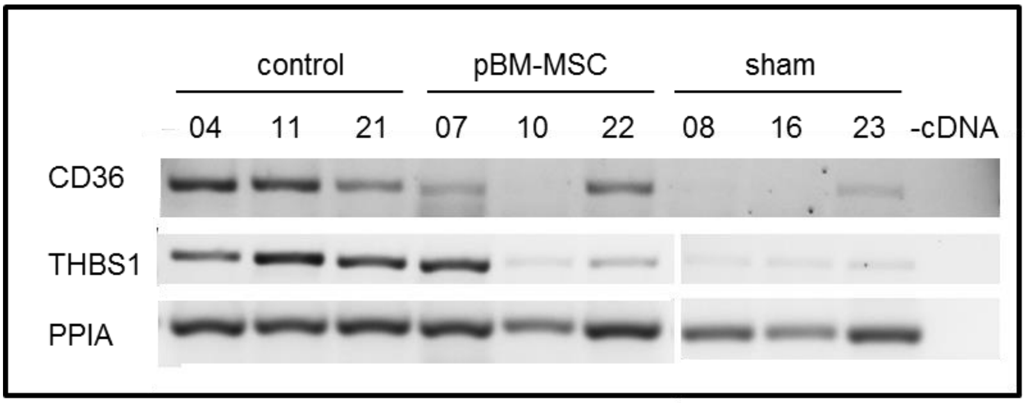

Supplementary Figure 2. MSC treatment attenuates the increase in CD36 and THBS1 mRNA levels after ePHx. Semiquantitative PCR analysis of tissue lysates from 3 different animals in each group. The representative electrophoretic gels shown here were used for semi-quantitative calculation of relative mRNA abundance as shown in Fig. 3c of the main manuscript. Figures above the gels delineate numbers of pigs used in the resp. experiment. Samples in each line were run on the same gel and were processed in parallel.

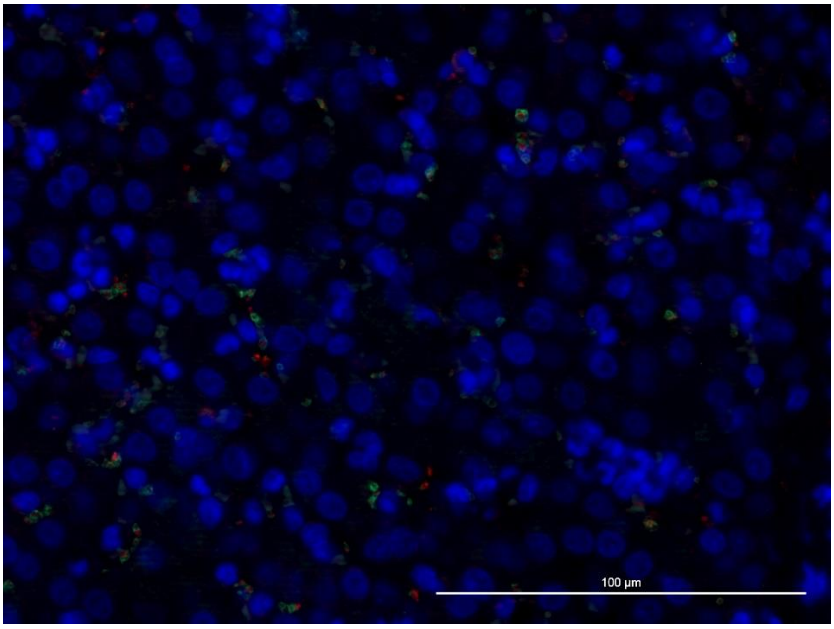

sham

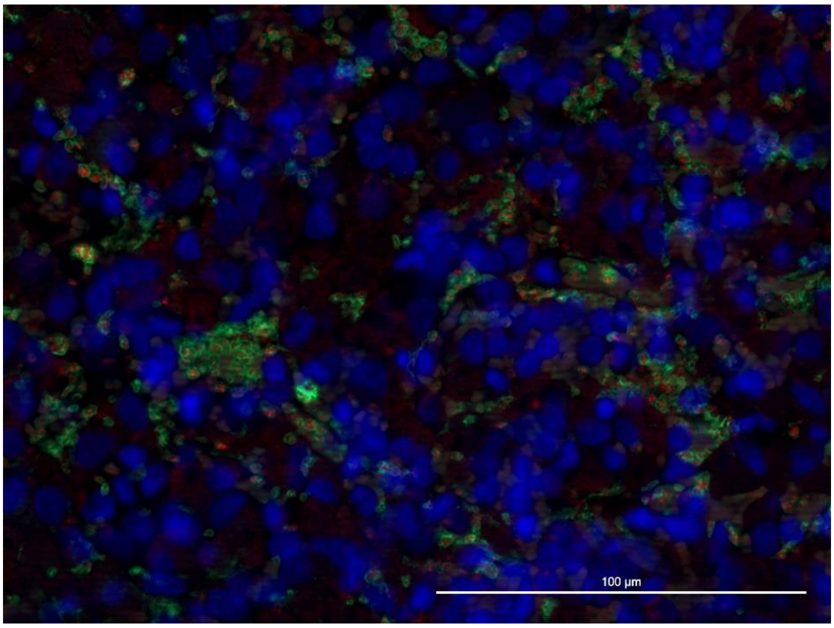

control

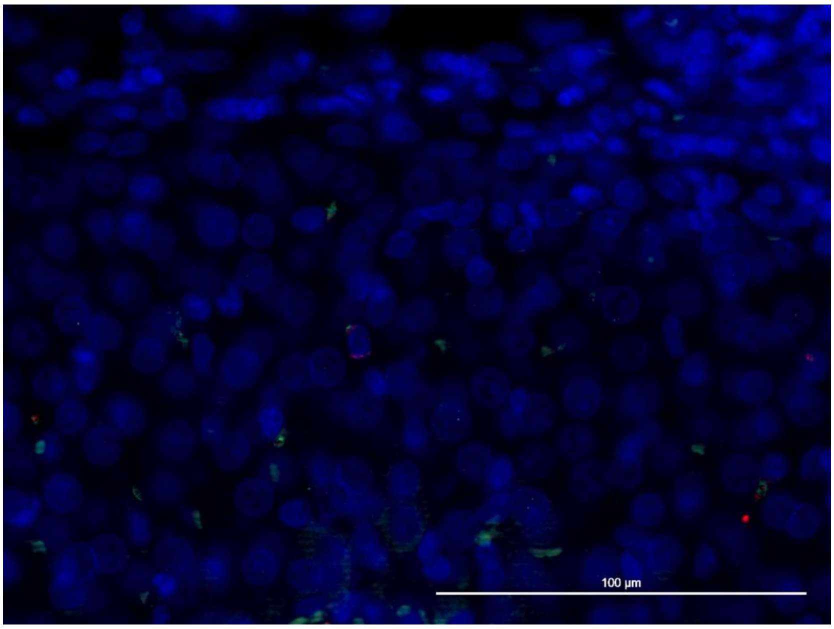

pBM-MSC

Supplementary Figure 3. Attenuation of the increase of platelets after ePHx in the liver by treatment with pBM-MSC. Thrombocytes in liver were visualized 24 h after ePHx by immunofluorescent detection of CD42b as a marker (green fluorescence). THBS1 (red fluorescence) co-localized with CD42b indicating thrombocytes as potential source of THBS1. As compared with sham-treated animals, ePHx markedly increased platelets in the liver (control), which was abrogated again by treatment with pBM-MSC. Original magnification – 40x. Scale bar – 100 µm.

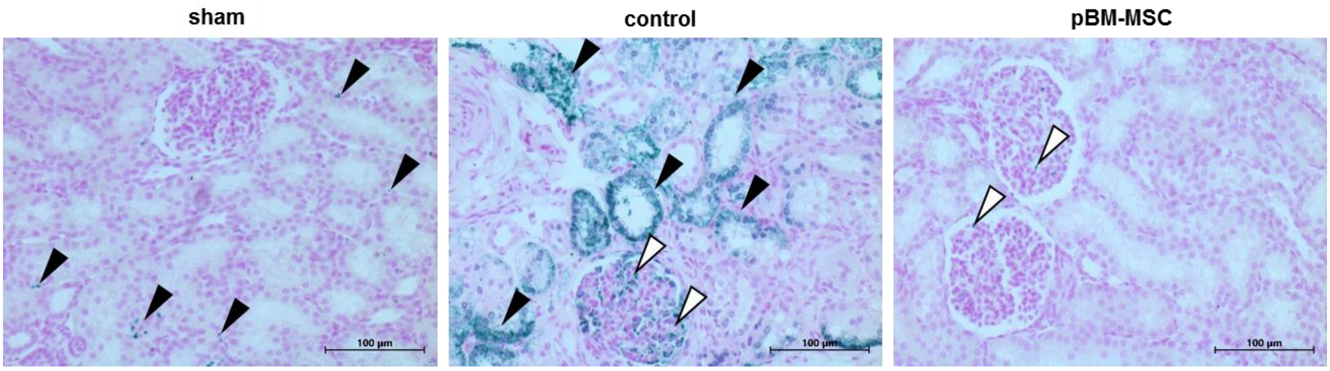

Supplementary Figure 4. Thrombocytes in the kidney after ePHx with and without MSC treatment. Immunohistochemical detection of thrombocytes using the CD42b antibody in kidneys of sham (left), control (middle), and MSC-treated (right) pigs at 24 h after liver surgery. Images show 20x original magnifications of representative organ tissue slices from 3 pigs per group. Black and white arrowheads indicate tubular and glomerular staining, resp. Scale bar – 100 µm.

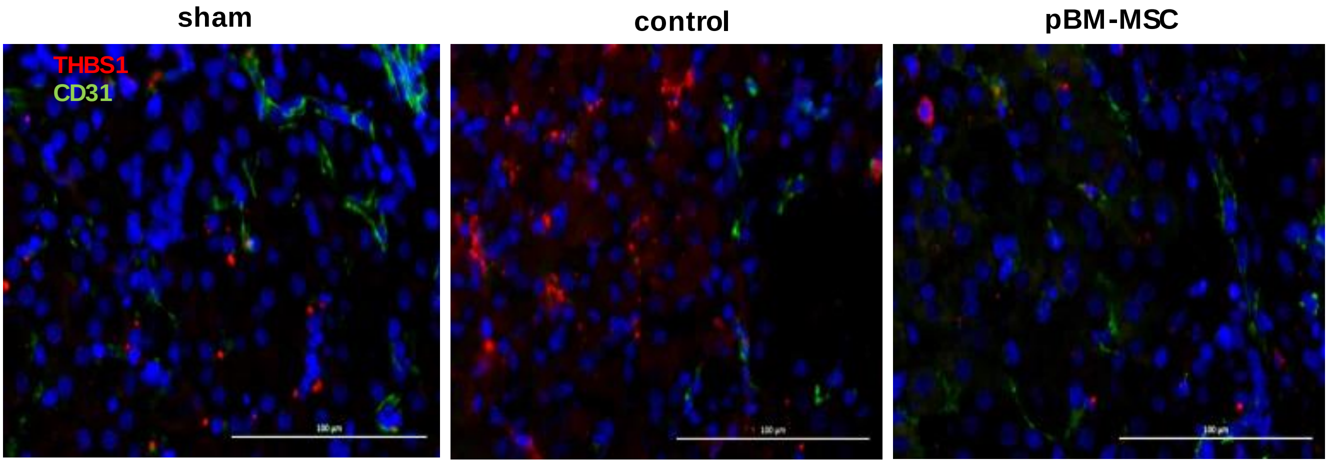

Supplementary Figure 5. THBS1 does not co-localize with CD31 on endothelial cells. THBS1was visualized 24 h after ePHx by immunofluorescent detection (red fluorescence). Endothelia were counterstained by detection of CD31 (green fluorescence). Original magnification – 20x. Scale bar – 100 µm.

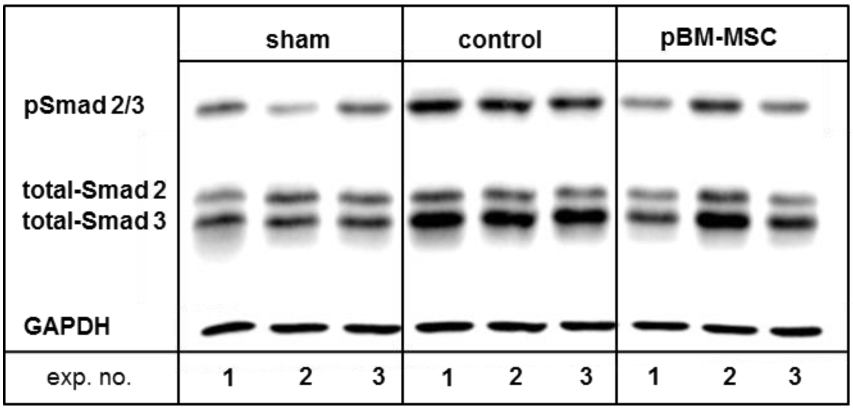

Supplementary Figure 6. MSC treatment attenuates the increase in pSmad 2/3 protein levels after ePHx. Western blot analysis of tissue lysates from 3 different animals in each group. The representative immunoblots shown here were used for semi-quantitative calculation of relative protein expression as shown in Fig. 5d of the main manuscript. Samples in each line were run on the same gel and processed in parallel.

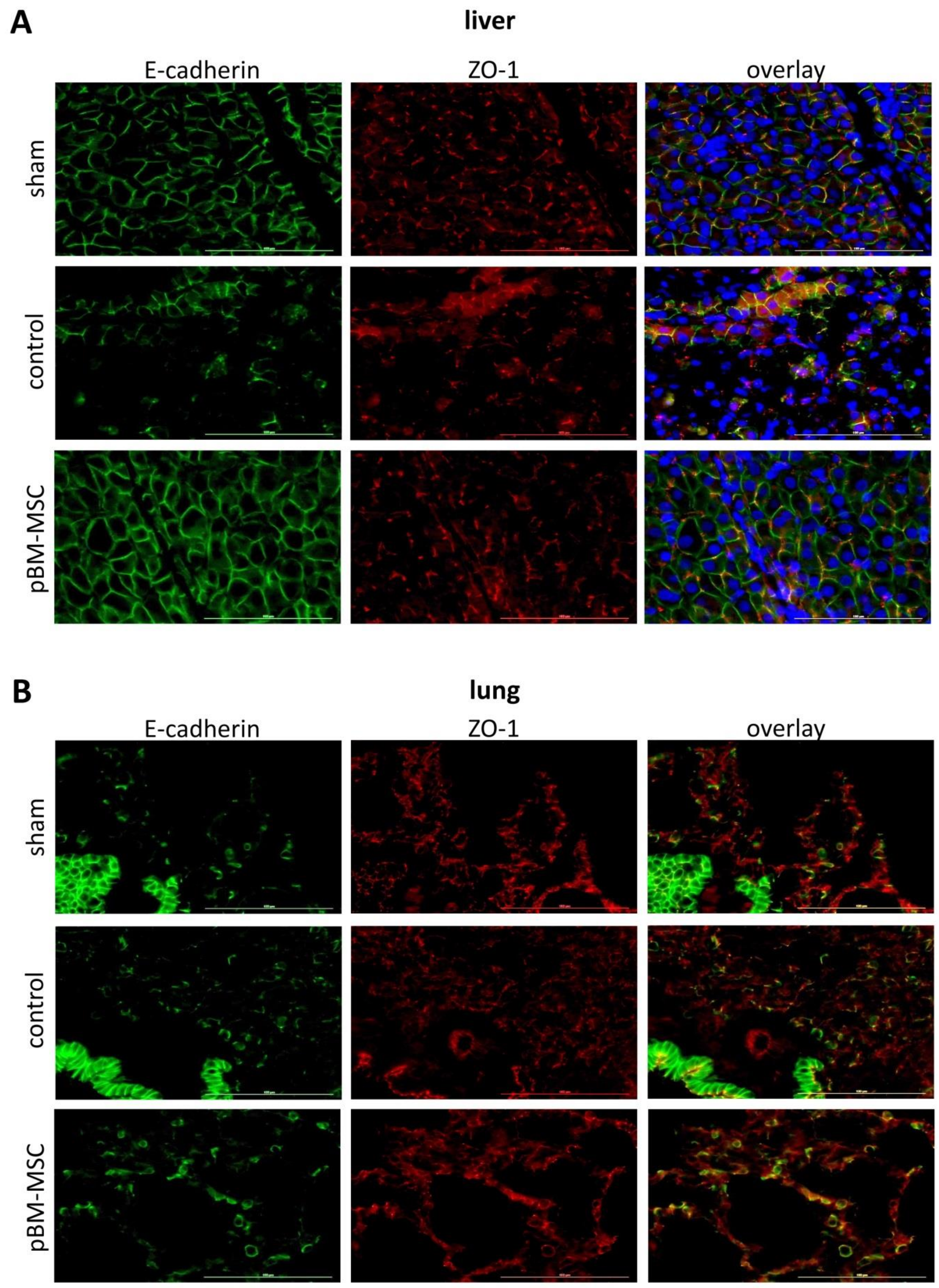

Supplementary Figure 7. MSC treatment protects tight junctions from damage after ePHx. Immunofluorescent detection of E-cadherin and ZO-1 in (A) liver and (B) lung of sham-, control-, and MSC-treated pigs; E-cadherin (left panels, green), ZO-1 (middle panels, red), and overlay with DAPI co-stains (right panels). Tissue samples were taken at 24 h after ePHx. In the livers, E-cadherin and ZO-1 fade away after resection (middle panels) in comparison with sham-treated animals (upper panels). In lung, ZO-1 features discontinuity

after liver resection. Both signs of tissue damage are attenuated by the treatment with pBM-MSC (middle vs. bottom panels). Images are representative for each 3 slices out of the organs from 3 animals per group. Original magnification – 20x.

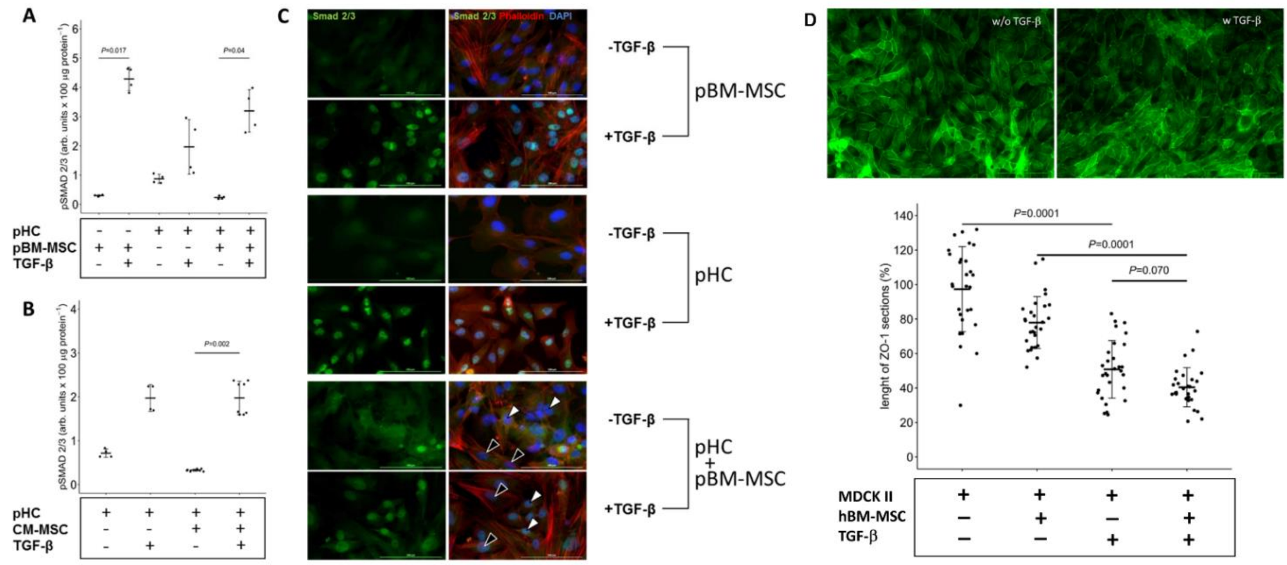

Supplementary Figure 8. pBM-MSC do not inhibit TGF-β signaling in primary porcine hepatocytes. (A) Hepatocytes and pBM-MSC were cultured alone or in combination (1:1). TGF-β (1 ng/mL) was included and pSmad 2/3 measured by ELISA. (B) Conditioned medium from pBM-MSC (CM-MSC) was used instead of pBM-MSC. In (C), TGF-β signaling was assessed in co-cultures of porcine hepatocytes and pBM-MSC by fluorescent immunocytochemical staining of nuclear translocation of Smad 2/3 using an antibody detecting the unphosphorylated Smad 2/3 (green). Actin was stained with phalloidin to facilitate detection of cells (red). pBM-MSC (black arrowheads) were discriminated from hepatocytes (white arrowheads) by differences in nuclear size (DAPI stain, blue) and actin staining. In the presence of TGF-β, nuclear translocation is observed in both single cultures and co-cultures. (D) hBM-MSC do not inhibit TGF-β action in MDCK II cells. MDCK II cells and hBM-MSC were co-cultured (1:1). MDCK II cells were treated or not with TGF-β (5 ng/mL) for 20 h in order to induce EMT as shown immunocytochemically by the diminution of membraneous ZO-1 (upper panels). For the quantification, the ZO-1 positive stain on microscopic pictures was manually delineated, and total lengths quantified using Image J. The length of ZO-1 contacts in cells without TGF-β was set to 100 %, to which values with TGF-β are relative. In each group, 28 different visual fields out of three independent cell culture experiments were used for calculations (lower panel). Values represent means±SEM and are significantly different at $P \leq 0.05$ as indicated (statistics: one-way ANOVA).

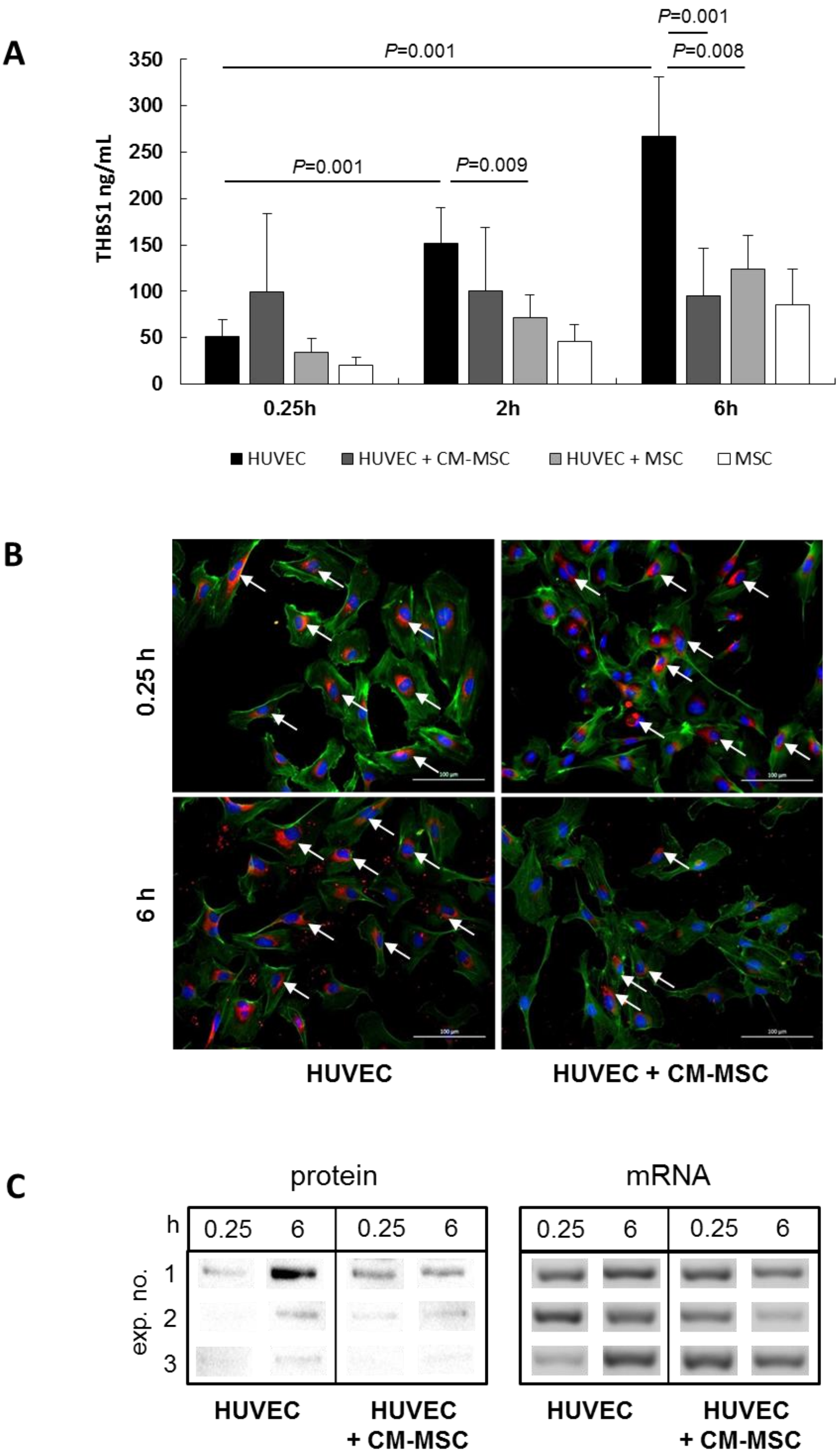

Supplementary Figure 9. pBM-MSC inhibit THBS1 secretion from HUVEC. (A) THBS1 secretion from HUVEC into the supernatant medium was determined by ELISA at the times indicated either in mono-cultures of HUVEC (HUVEC) and MSC (MSC), or in co-culture of both (HUVEC+MSC), or in conditioned medium derived from MSC (HUVEC+CM-MSC). Values are means±SEM (n=6; statistics: one-way ANOVA after logarithmic transformation of the dataset) and are significantly different at the *P*-levels as indicated. (B) THBS1 was visualized in HUVEC by immunofluorescence (red, arrows). The actin cytoskeleton was visualized with phalloidin (green), and nuclei with DAPI (blue). (C) THBS1 expression was

evaluated both on the protein and mRNA level by Western blotting and RT-PCR. The blots show results from 3 different independent cell culture experiments. Lysates to isolate protein and RNA were prepared from the HUVEC at two different time-points (0.25 and 6 h of culture) of treatment with or without MSC-derived conditioned medium (CM-MSC). Samples in each line were run on the same gel and processed in parallel.

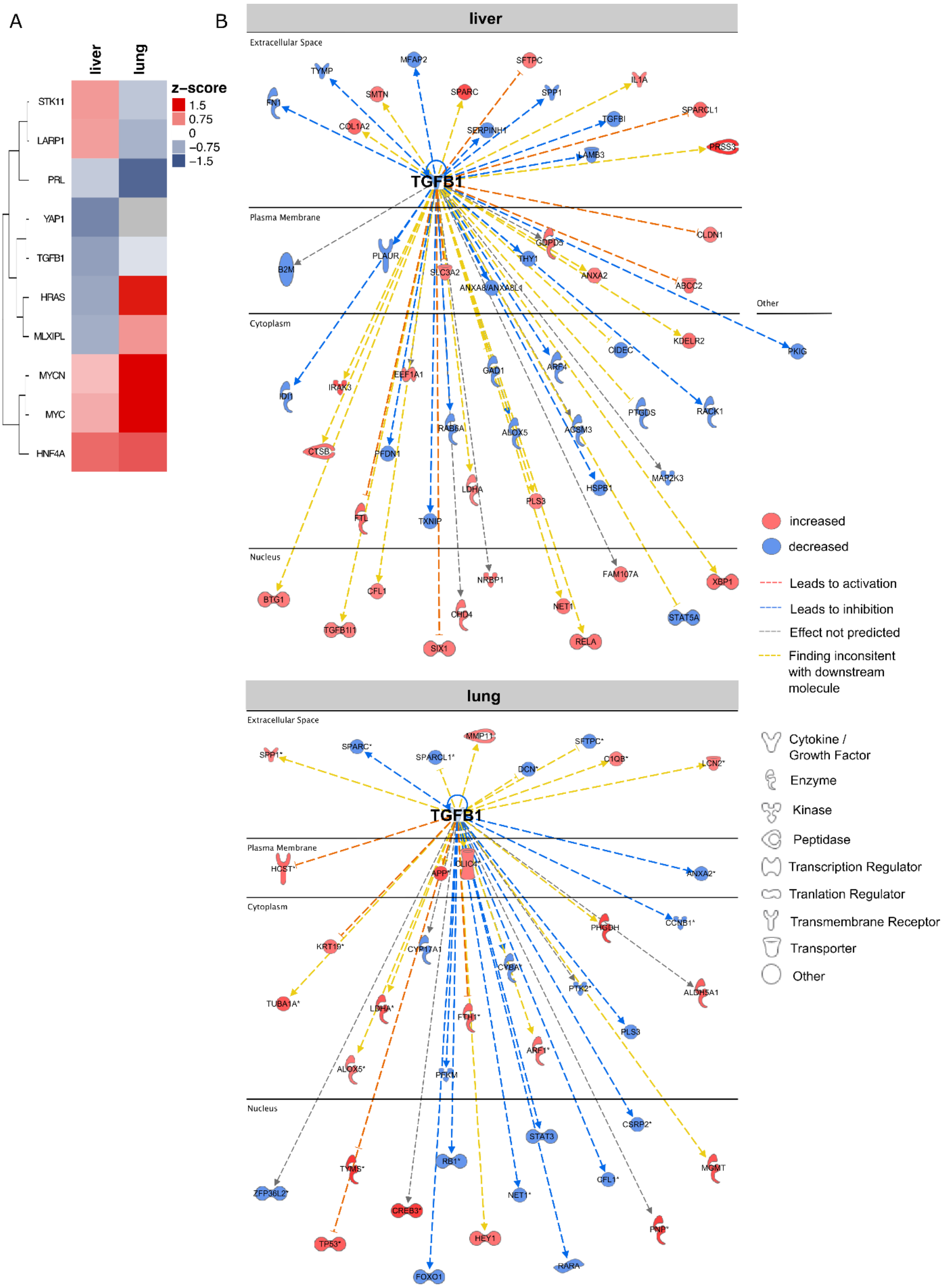

Supplementary Figure 10. Upstream regulator analysis yielding pathways affected by MSC treatment in liver and lung. (A) The Top 10 significantly enriched upstream

regulators (P-value $\leq$0.05) affected by MSC-treatment in liver and lung were predicted *in silico* using Ingenuity Pathway Analysis. (B) The regulatory network of TGF-β target genes in liver and lung were visualized based on their cellular compartments.

Supplementary Methods

Surgical procedure

Adult male German landrace pigs were obtained from the farm product company Kitzen (Pegau, Germany) and were housed at the Experimental Centre of the Faculty of Medicine, University of Leipzig, under a 12 h circadian rhythm at 25°C receiving a standard pig diet for at least 3 days. Animals were starved 24 h before surgery, and underwent a veterinary inspection verifying body weight (25-30 kg), temperature (below 40°C), and general health condition. They received a premedication (ketamine 15 mg/kg BW, atropine 0.01 mg/kg BW, and midazolam 0.5 mg/kg BW) to facilitate transportation into the operating theatre. A venous catheter was placed in the dorsal *V. auricularis* (Supplementary Figure 11-1) and total venous anesthesia and analgesia (midazolam 1 mg/kg BW/h, sufentanil 0.5 µg/kg BW/h, ketamine 5-15 mg/kg BW/h, pancuronium bromide 80 µg/kg BW/h) was administered through the catheter. After fixing the sedated animal onto the operating table (Supplementary Figure 11-2), a tracheotomy (Supplementary Figure 11-3) for mandatory ventilation using a respiration apparatus (type Julian, Drägerwerk AG & Co. KGaA, Lübeck, Germany) was performed (Supplementary Figure 11-4). In addition, a central venous catheter was fixed in the *V. jugularis interna* (Supplementary Figure 11-5) for the central venous application of the total anesthesia and analgesia medication. The catheter served also for infusion of control solution (Ringer´s acetate, Gelafusal® (Serumwerk Bernburg AG, Bernburg, Germany), and dextrose 5%; 6:3:1) in the control, and pBM-MSC transfusion in the treatment groups. The abdominal cavity was opened by an incision along the *linea alba* (60 cm) (Supplementary Figure 11-6+7). Liver lobes were mobilized (Supplementary Figure 11-8), and portocaval anastomosis (Supplementary Figure 11-9) was established to enable the gastrointestinal blood bypass of

the liver. The *ligamentum hepatoduodenale* was clamped (Supplementary Figure 11-10) to stop the venous and arterial blood flow into the liver (Pringle's manoeuvre). 70% of the total liver mass were removed by resecting the left and medial liver lobes (Supplementary Figure 11-11). Coagulation of the resection areas was achieved using bipolar forceps (Supplementary Figure 11-12) and hemostyptics (TABOTAMP Fibrillar, Johnson & Johnson GmbH, Norderstedt, Germany). After a total time of 150 min warm ischemia, the portocaval anastomosis was occluded (Supplementary Figure 11-13) and the clamping of the *Lig. hepatoduodenale* was released. Then, the abdominal cavity was closed (Supplementary Figure 11-14) and pBM-MSC or Ringer´s solution (6:3:1 Ringers acetate, Gelafusal® (Serumwerk Bernburg AG, Bernburg, Germany), and dextrose 5%) were infused. All animals were subjected to intensive care monitoring and treatment for 24 h. To maintain cardiovascular performance as well as glucose homeostasis, animals were supplemented with Norepinephrine and glucose as appropriate. After 24 h, animals were euthanasied by a lethal dose of intravenous anesthesia and analgesia medication in addition to intravenous injection of potassium chloride solution. Organs (lung (Supplementary Figure 11-15), liver remnant (Supplementary Figure 11-16)) were harvested at the end of the experiment.

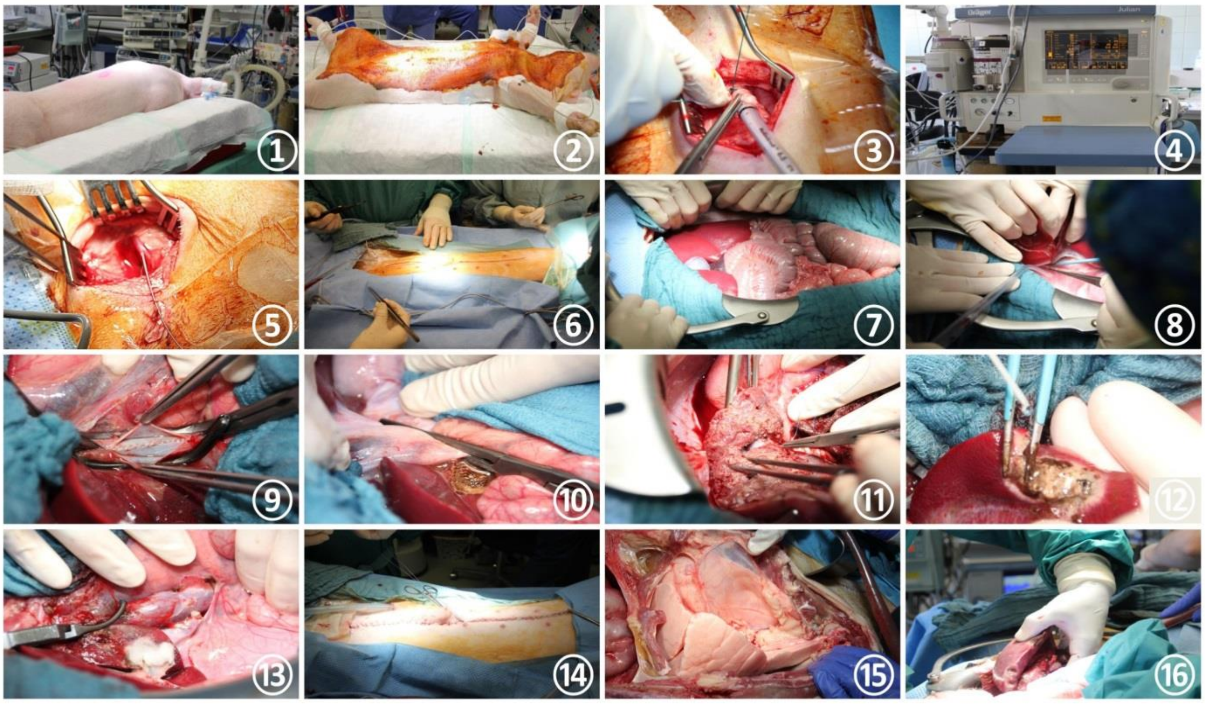

Supplementary Figure 11. Surgical procedure of extended liver resection in the pig. (1) Positioning of the venous catheter in the dorsal V. auricularis for total intravenous anesthesia. (2) Fixation of the sedated animal onto the operating table. (3) Tracheotomy. (4) Mandatory ventilation apparatus. (5) Cannulation of the V. jugularis interna for positioning of the central venous catheter placement. (6) Incision along the linea alba and (7) positioning of retractor. (8) Mobilization of liver lobes. (9) Portocaval anastomosis using 6-0 monofile non resorbable sutering material in a running suture technique. (10) Occlusion of the ligamentum hepatoduodenale (Pringle's manoeuvre). (11) Resection of the left and medial liver lobes. (12) Coagulation of resection surface. (13) Occlusion of the portocaval anastomosis after 150 min warm ischemia time. (14) Closure of the abdominal cavity. (15) Harvest of lung and (16) remnant liver.

Supplementary Table 1. Summary of antibodies and dilutions used in this study.

Antibody	Species	Dilution	Cat. No.	Supplier
**Immunohistochemistry**				
E-cadherin	mouse	1:200	610182	BD, Heidelberg, Germany
N-cadherin	rabbit	1:200	04-1126	Millipore, Darmstadt, Germany
ZO-1	rabbit	1:150	44-2200	InVitrogen, Carlsbad, CA, USA
CD31	rabbit	1:75	ab28364	Abcam, Cambridge, England
CD42b	rabbit	1:200	ab183345	Abcam, Cambridge, England
α-SMA	rabbit	1:250	ab5694	Abcam, Cambridge, England
THBS1	mouse	1:100	ab1823	Abcam, Cambridge, England
Smad2/3	rabbit	1:500	8485S	Cell Signaling, Cambridge, England
TGF-β	rabbit	1:200	bs4538R	Fisher-Scientific, Waltham, MA, USA
Heparan sulfate	mouse	1:200	ab23418	Abcam, Cambridge, England
Cy3-labeled anti-mouse	goat	1:200	115-165-003	Dianova, Hamburg, Germany
AlexaFluor 488-labeled anti-rabbit	goat	1:200	A11008	Life Technologies, Carlsbad, CA, USA
Biotin-labeled anti-mouse	goat	1:200	115-065-003	Dianova, Hamburg, Germany
Biotin-labeled anti-rabbit	donkey	1:400	711-065-152	Dianova, Hamburg, Germany
HRP-labeled anti-rabbit	goat	1:200	554021	BD, Heidelberg, Germany
**Immunocytochemistry**				
Smad 2/3	rabbit	1:500	8485S	Cell Signaling, Cambridge, England
THBS1	mouse	1:100	ab1823	Abcam, Cambridge, England

ZO-1	rabbit	1:100	44-2200	InVitrogen, Carlsbad, CA, USA
Phalloidin iFluor 488		1:500	ab176753	Abcam, Cambridge, England
Cy3-labeled anti-mouse	goat	1:200	115-165-003	Dianova, Hamburg, Germany
AlexaFluor 488-labeled anti-rabbit	goat	1:200	A11008	Life Technologies, Carlsbad, CA, USA
**Western blot**				
E-cadherin	mouse	1:2000	610182	BD, Heidelberg, Germany
ZO-1	rabbit	1:700	44-2200	InVitrogen, Carlsbad, CA, USA
N-cadherin	rabbit	1:2000	04-1126	Millipore, Darmstadt, Germany
Smad 2/3	rabbit	1:1000	8685S	Cell Signaling, Cambridge, England
pSmad 2/3	rabbit	1:1000	8828S	Cell Signaling, Cambridge, England
THBS1	mouse	1:100	ab1823	Abcam, Cambridge, England
Vimentin	mouse	1:1000		Abcam, Cambridge, England
α-SMA	rabbit	1:2000	ab5694	Abcam, Cambridge, England
Vinculin	mouse	1:1000	05-386	Merck, Darmstadt, Germany
GAPDH	mouse	1:3000	NB300-221	Novusbio, Abingdon, England
HRP-labeled anti-mouse	goat	1:5000	554002	BD, Heidelberg, Germany
HRP-labeled anti-rabbit	goat	1:5000	554021	BD, Heidelberg, Germany

Supplementary Table 2. Summary of primer pairs used for semi-quantitative RT-PCR.

Gene	Ref seq	Fwd 5‘→3‘	Rev 5‘→3‘
CD36	NM_001044622.1	TAATGAGACTGGTACAATTGGTGAT	TCATGATGGTAGTGATCTTTTCTCC
THBS1 (thrombo-spondin-1)	NM_001244536.1	ATCTCCGCATCGCAAAAGGA	ATTGGTAGAGCTGGAGCAACC
PPIA (peptidylprolyl isomerase A)	NM_214353.1	GTCTTCTTCGACATCGCCGT	GCACGGAAGTTTTCTGCTGTC

Supplementary Table 3. Summary of proteins detected by the Proteome Profiler Human XL Cytokine Array Kit

Adiponectin	FGF-19	IL-18BPa	PDGF-AA
Aggregan	Flt-3 Ligand	IL-19	PDGF-AB/BB
Angiogenin	G-CSF	IL-22	Pentraxin-3
Angiopoietin-1	GDF-15GM-CSF	IL-17A	Osteopontin
Angiopoietin-2	GRO-alpha	IL-23IL-24	PF4
BAFF	Growth Hormone	IL27	RAGE
BDNF	HGF	IL-31	RANTES
Complement Component C5/C5a	ICAM-1	IL-32alpha/ß/gamma	RBP4
CD14	INF-gamma	IL-33	Relaxin-2
CD30	IGFBP-2	IL-34	Resistin
CD40 ligand	IGFBP-3	IP-10	SDF-1alpha
Chitinase 3-like 1	IL-1alpha	I-TAC	Serpin E1
Complement Factor D	IL-1ß	kallikrein 3	SHBG
CRP	IL-1ra	Leptin	ST2
Cripto-1	IL-2	LIF	TARC
Cystatin C	IL-3	Lipocalin-2	TFF3
Dkk-1	IL-4	MCP-1	TfR
DPPIV	IL-5	MCP-3	TGF-alpha
EGF	IL-6	M-CSF	Thrombospondin-1
EMMPRIN	IL-8	MIF	TNF-alpha
ENA-78	IL-10	MIG	uPAR
Endoglin	IL-11	MIP-1alpha/MIP-1ß	VEGF
Fas Ligand	IL-12p70	MIP-3alpha	Vitamin D BP
FGF basic	IL-13	MIP-3ß	
FGF-7	IL-15	MMP-9	
	IL-16	Myeloperoxidase	
